# Penicillin-Binding Protein 5/6 Acting as a Decoy Target in Pseudomonas aeruginosa Identified by Whole-Cell Receptor Binding and Quantitative Systems Pharmacology

**DOI:** 10.1128/aac.01603-22

**Published:** 2023-05-18

**Authors:** Silvia López-Argüello, Maria Montaner, Alaa RM. Sayed, Antonio Oliver, Jürgen B. Bulitta, Bartolome Moya

**Affiliations:** a Servicio de Microbiología and Unidad de Investigación, Hospital Universitario Son Espases, Instituto de Investigación Sanitaria Illes Balears (IdISBa), Palma de Mallorca, Spain; b Department of Pharmacotherapy and Translational Research, College of Pharmacy, University of Florida, Orlando, Florida, USA; c Department of Chemistry, Faculty of Science, Fayoum University, Fayoum, Egypt

**Keywords:** Penicillin binding proteins (PBP), intact cells, outer membrane penetration, bacterial permeability, beta-lactams/beta-lactamase inhibitors, quantitative and systems pharmacology (QSP)

## Abstract

The β-lactam antibiotics have been successfully used for decades to combat susceptible Pseudomonas aeruginosa, which has a notoriously difficult to penetrate outer membrane (OM). However, there is a dearth of data on target site penetration and covalent binding of penicillin-binding proteins (PBP) for β-lactams and β-lactamase inhibitors in intact bacteria. We aimed to determine the time course of PBP binding in intact and lysed cells and estimate the target site penetration and PBP access for 15 compounds in P. aeruginosa PAO1. All β-lactams (at 2 × MIC) considerably bound PBPs 1 to 4 in lysed bacteria. However, PBP binding in intact bacteria was substantially attenuated for slow but not for rapid penetrating β-lactams. Imipenem yielded 1.5 ± 0.11 log_10_ killing at 1h compared to <0.5 log_10_ killing for all other drugs. Relative to imipenem, the rate of net influx and PBP access was ~ 2-fold slower for doripenem and meropenem, 7.6-fold for avibactam, 14-fold for ceftazidime, 45-fold for cefepime, 50-fold for sulbactam, 72-fold for ertapenem, ~ 249-fold for piperacillin and aztreonam, 358-fold for tazobactam, ~547-fold for carbenicillin and ticarcillin, and 1,019-fold for cefoxitin. At 2 × MIC, the extent of PBP5/6 binding was highly correlated (*r*^2^ = 0.96) with the rate of net influx and PBP access, suggesting that PBP5/6 acted as a decoy target that should be avoided by slowly penetrating, future β-lactams. This first comprehensive assessment of the time course of PBP binding in intact and lysed P. aeruginosa explained why only imipenem killed rapidly. The developed novel covalent binding assay in intact bacteria accounts for all expressed resistance mechanisms.

## INTRODUCTION

Multidrug-resistant Pseudomonas aeruginosa has been a relentless threat during the last decades. This microorganism is one of the leading causes of nosocomial infections with high rates of poor clinical outcomes and mortality (1–3). To combat P. aeruginosa, β-lactam antibiotics are often used in mono- and combination therapies, owing to their activity against susceptible P. aeruginosa and excellent safety. Despite the abundant clinical use of β-lactams for 8 decades, there is a nearly complete dearth of data on the extent and time course of penicillin-binding protein (PBP) receptor binding in intact P. aeruginosa and other pathogens.

Bacteria have multiple different PBPs, which serve different biochemical functions. In P. aeruginosa, the high molecular weight PBPs 1a and 1b have both transpeptidation and transglycosylation capabilities, with the former being inhibited by β-lactams (4, 5). The PBP2 and PBP3 are also categorized as high molecular weight PBPs, and they perform transpeptidation (but not transglycosylation) relating to cell elongation for PBP2 and cell division for PBP3. Among the lower molecular weight PBPs 4, 5/6, and 7, the PBP5/6 is the highest expressed PBP, and PBP4 inactivation has been shown to rapidly upregulate the AmpC β-lactamase in P. aeruginosa (6). Therefore, for β-lactams that are susceptible to AmpC-related hydrolysis, not inactivating PBP4 is beneficial. Instead, targeting the high molecular weight PBPs 1a, 1b, 2, and 3 are considered essential, but their extract contributions to bacterial killing have not been fully elucidated.

Each β-lactam binds to 1 or multiple different PBPs with different affinities via a covalent bond (4). The active site of PBPs is highly conserved in Gram-negatives. Before a β-lactam can bind to a PBP target receptor, it must penetrate through the outer membrane (OM) of Gram-negatives as well as avoid being hydrolyzed by β-lactamases or effluxed (7, 8). The OM of Gram-negative pathogens represents a formidable penetration barrier for β-lactams and many other antibiotics (9), especially in P. aeruginosa and Acinetobacter baumannii (10, 11).

The impact of OM permeability is highlighted by comparing the PBP binding in lysed cells with the MICs and rates of bacterial killing. While carbapenems have comparable PBP binding patterns (i.e., IC_50_s) in lysed cells, they differ considerably in their MICs and rates of bacterial killing (12–14). This has been linked to different β-lactam OM penetration rates in Escherichia coli (15). The PBP binding assays in lysed bacteria (i.e., using isolated membrane fractions either with radioactive [iodine-131] β-lactam probes or with Bocillin FL [Boc-FL]) provide valuable insights on the preferred PBP targets of each β-lactam (16–19); and lysed cell assays have been widely applied in E. coli and P. aeruginosa (20). However, this lysed cell assay does not account for OM penetration, access to different PBPs in periplasm, and efflux (16, 17, 21, 22). Studying the time course of PBP binding in intact bacteria offers the substantial advantage that it allows one to determine the rate of net influx and PBP access for different β-lactam molecules. There is a competition between influx and loss of β-lactam molecules due to efflux or β-lactamase-related hydrolysis in periplasm. Additionally, each β-lactam molecule binds to and thereby inactivates 1 PBP target and therefore there is an additional competition for the access to multiple different PBPs. Due to the covalent (and pseudo-irreversible) binding of PBPs, this assay accounts for all expressed resistance mechanisms.

In P. aeruginosa, a few studies quantified the OM permeability for a limited number of β-lactams via the Zimmermann-Rosselet (10, 11, 23–26) assay using spectrophotometric methods, as opposed to contemporary LC-MS/MS (27). Other studies used cell morphology changes as a downstream consequence of PBP inactivation by β-lactams that inhibit 1 or multiple PBPs but did not directly assess PBP binding in whole cells (28–30). Only a few recent publications have studied PBP binding using a whole-cell assay at a single time point and used strains with normal efflux pump and AmpC β-lactamase function (12, 19, 31). These studies neither assessed the rate of target site penetration nor modeled the PBP binding. Two of these studies aimed to establish different fluorescent probes in E. coli or to optimize a prototype whole-cell binding assay in A. baumannii (12, 19). In P. aeruginosa, the complex interplay between OM permeability and resistance mechanisms may have contributed to the lack of observed correlations between β-lactam structure, PBP inhibition profiles, MICs, and killing kinetics (31). Despite these considerable gaps in the mechanisms of β-lactam action in Gram-negatives, 13 randomized controlled clinical trials in the 1970s and 1980s yielded promising results for empirical non-optimized double β-lactam combination therapies (32). This strongly suggests that optimized double β-lactam combinations are clinically viable, especially if they are mechanistically optimized.

In this work, our primary aim was to systematically characterize the time course of covalent PBP binding by 15 structurally diverse β-lactams and β-lactamase inhibitors (BLIs) in intact and lysed P. aeruginosa. Secondly, we aimed to estimate the rate of net influx and PBP access based on differences in the time course of PBP binding between intact and lysed cells via a novel Quantitative and Systems Pharmacology (QSP) model. For the first time, this model implemented mass balance equations in periplasm and accounted for the relative expression of PBPs. This approach allowed us to characterize the rate and extent of receptor binding at the periplasmic target site and to thereby provide guidance for rationally optimizing β-lactam therapies and drug development. We found that the most highly expressed PBP5/6 acted as a decoy target (i.e., a sink) that should be avoided during the development of slowly penetrating, future PBP-binders.

## RESULTS

### Relative PBP expression.

**FIG 1:**
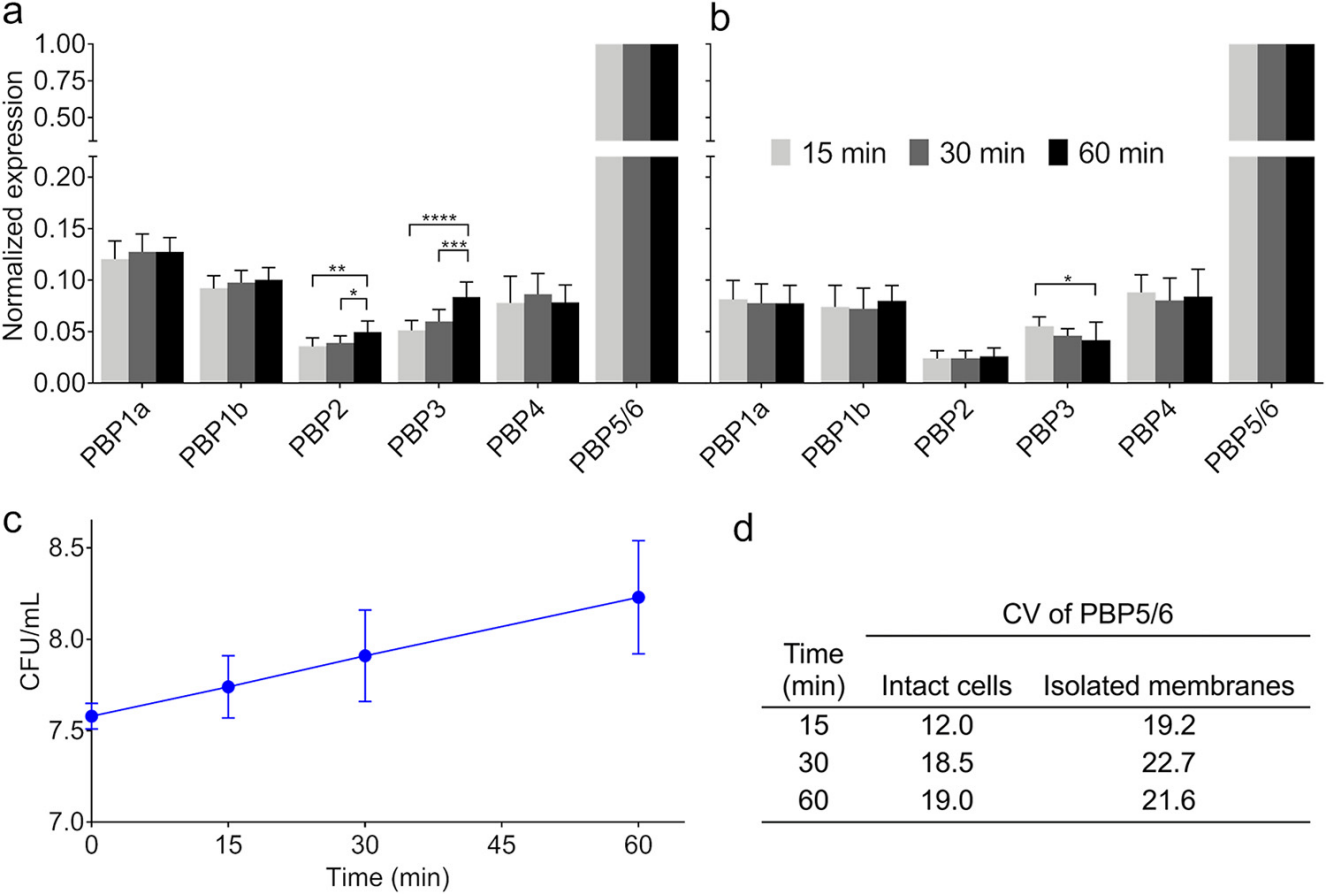
Normalized protein expression and relative band intensity of the whole cell and isolated membrane PBP binding assays. Normalized PBP expression relative to PBP5/6 in (a) whole cells and (b) isolated membranes. The columns represent the average PBP expression values obtained from at least 3 biological replicates, and the error bars show the standard deviations. One-way ANOVA with *post hoc* Tukey’s multiple comparison test was run to determine the statistically significant differences over time (*, *P*-value < 0.05; **, *P*-value < 0.01; ***, *P*-value < 0.001; ****, *P*-value < 0.0001). Bacterial growth (CFU/mL) in the whole cells assay (c). The average viable counts from at least 3 experiments ± standard deviations are shown. The data were normalized to the highest expression PBP, which was always PBP5/6. Therefore, PBP5/6 contained no error bars. (d) Coefficient of variation (CV) of PBP5/6 expression across the 3 replicates.

To determine the relative PBP expression, the band intensity values relative to that of PBP5/6 (i.e., the most abundant PBP in P. aeruginosa [16]), were determined in the untreated control at 0, 15, 30, and 60 min. The PBP5/6 was by far the highest expressed PBP, followed by PBP1a, PBP1b, and PBP4 (Fig. 1a and b). The signals of PBP3 and PBP2 were 18 to 78-fold lower than that of PBP5/6.

In intact cells, a steady increase in expression by ~ 20% over the first 60 min was observed for PBP2 and by ~ 40% for PBP3 (Fig. 1a). This increased expression was correlated with bacterial growth (i.e., log_10_ CFU/mL; *r*_PBP2_ = 0.84, *r*_PBP3_ = 0.91). In contrast, the expression of PBP1a, 1b, 4, and 5/6 was relatively constant during 60 min. For lysed cells, we isolated membrane fractions and then incubated them for 15, 30, or 60 min at 37°C. During this incubation, PBPs were stable and only PBP3 showed a small decline (Fig. 1b).

### Time-course PBP binding in whole cells.

**FIG 2:**
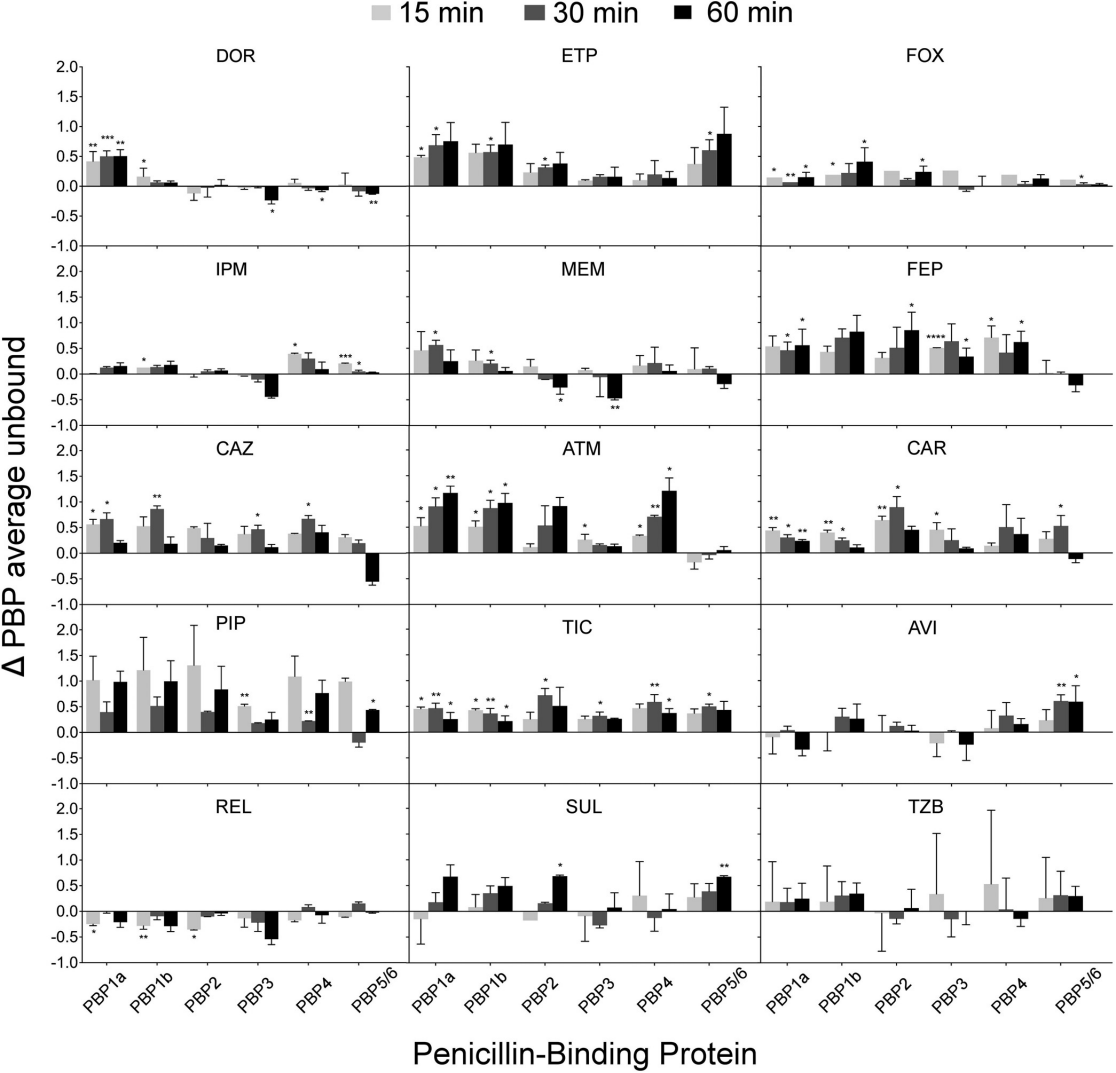
Difference between whole cells and isolated PBPs fraction unbound. P. aeruginosa PAO1 cultures (whole cells) and PBP-containing membrane preparations (isolated) were incubated for 15, 30, and 60 min in the presence of doripenem (DOR); ertapenem (ETP); imipenem (IPM); meropenem (MEM); cefepime (FEP); cefoxitin (FOX); ceftazidime (CAZ); aztreonam (ATM); carbenicillin (CAR); piperacillin (PIP); ticarcillin (TIC); avibactam (AVI); relebactam (REL); sulbactam (SUL); tazobactam (TZB), and afterwards isolated PBPs labeled with 25 μM Boc-FL. The resulting 0.5 mg/mL of PBP-containing membrane preparations (whole cells) were labeled with 25 μM Boc-FL. The antibiotic concentrations tested were 2 × MIC: DOR = 2 mg/L; ETP = 8 mg/L; IPM = 2 mg/L; MEM = 1 mg/L; FEP = 2 mg/L; FOX = 2,048 mg/L; CAZ = 2 mg/L; ATM = 8 mg/L; CAR = 96 mg/L; PIP = 8 mg/L; TIC = 48 mg/L. The BLIs AVI, REL, SUL, and TZB were used at a fixed concentration of 4 mg/L. Data represent the difference between average fraction of unbound PBPs in the whole-cell and isolated membrane assays from at least three biological replicates; the error bars show the SD of the differences. Unpaired Student’s *t* tests were applied to determine statistically significant differences (*, *P*-value < 0.05; **, *P*-value < 0.01; ***, *P*-value < 0.001; ****, *P*-value < 0.0001).

**FIG 3:**
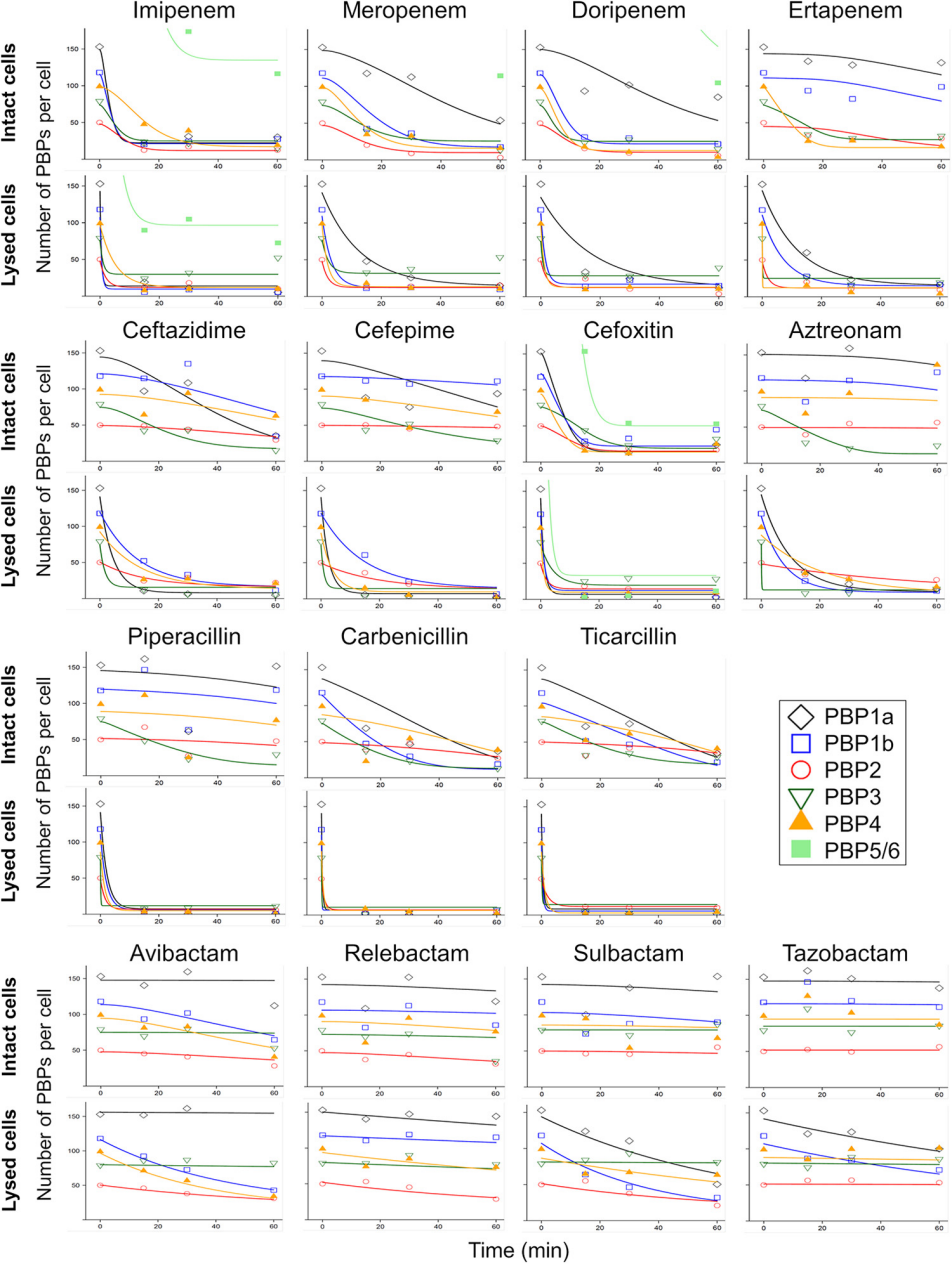
Time-course of PBP binding for PBPs 1a, 1b, 2, 3, 4, and 5/6 in intact (upper rows) and lysed cells (lower rows) for 15 drugs. The markers are the means of the observations, and the lines are the curve-fits from simultaneous modeling of the PBP binding data from QSP modeling. This plot focused (i.e., zoomed in) on the most important PBPs, and the same data with the full *y* axis range (due to PBP5/6) is shown in the supplement (Fig. S7).

Control experiments with Boc-FL exposure durations between 5 and 30 min showed no significant difference and thus 15 min was used as final Boc-FL exposure duration. Only imipenem showed an approximately 1.5 log_10_ reduction in viable counts during the first 60 min, whereas all other β-lactams yielded <0.5 log_10_ killing (Fig. S1). This suggested rapid penetration and PBP binding by imipenem. In the intact cell PBP binding assay with β-lactams at 2 × MIC, imipenem (2 mg/L) and cefoxitin (2,048 mg/L) rapidly inactivated all PBPs. In contrast, target site penetration and PBP inactivation was slightly slower for doripenem and meropenem, and substantially slower for ertapenem (Fig. 2 and 3, and Fig. S2 and S4) in whole cells. Aztreonam highly selectively inactivated PBP3, whereas ceftazidime and cefepime additionally inactivated PBP1a and, to some extent, PBP4. Ceftazidime showed considerable binding of PBP5/6 at 60 min, but not at earlier time points, which may have been an ‘outlier’ that could not be explained by QSP modeling (Fig. S7). The penicillin inactivated multiple PBPs, although slower than carbapenems. The BLI showed slow and limited PBP inhibition at 4 mg/L.

### Time-course PBP binding in lysed cells.

All carbapenems (including ertapenem) and cefoxitin showed rapid and extensive PBP binding (Fig. 3, and Fig. S3 and S5). There was slightly more noise for PBP3 binding by imipenem and meropenem with the fraction of unbound PBPs increasing slightly from 30 to 60 min (Fig. 3 and Fig. S5). This unexpected observation did not occur for ertapenem and was explained by a slightly larger background noise via QSP modeling (Table S2). The penicillin relatively rapidly inactivated all PBPs except PBP5/6. Cefepime, ceftazidime, and aztreonam also lacked PBP5/6 binding and slowly bound PBP2. In the lysed cell assay, 8 mg/L aztreonam (i.e., 2 × MIC) inactivated both the primary target PBP3 as well as PBP1a and PBP1b. The PBP binding by avibactam and sulbactam was relatively slow, and more extensive than PBP binding by relebactam or tazobactam.

### Extent of PBP binding.

When comparing the extent of PBP binding over the first 60 min between intact and lysed cells (Fig. 2), imipenem, doripenem, and meropenem showed extensive inactivation with only a few significant differences between intact and lysed cell PBP inactivation (Fig. 2). A positive (negative) number for the ΔPBP indicated less (more) PBP binding in intact compared to that in lysed cells. Imipenem showed ΔPBP close to zero, demonstrating rapid penetration. The ΔPBP were slightly positive for doripenem, meropenem, and cefoxitin (at 2,048 mg/L), but were substantially positive for ertapenem, demonstrating that ertapenem penetration was slowest among the carbapenems. Ceftazidime, ticarcillin, and carbenicillin showed moderately positive ΔPBP, whereas the ΔPBP were larger for cefepime and largest for piperacillin and aztreonam, suggesting poor penetration. With the caveat that the extent of PBP binding was limited for the BLIs, the ΔPBP for avibactam were small suggesting a reasonable penetration.

When comparing the overall extent of PBP inactivation (based on the AUC_0-60min_), imipenem, doripenem, and meropenem achieved extensive binding with only a few significant differences between intact and lysed cells (Table 1). This suggested rapid penetration of these 3 carbapenems in intact cells. While ertapenem yielded extensive binding of all PBPs in lysed cells, it did not significantly bind PBP1a and PBP5/6 in intact cells, suggesting comparatively poor penetration. Ceftazidime showed more extensive PBP inactivation in intact cells than cefepime, suggesting a slightly faster penetration of ceftazidime. Aztreonam only bound its primary target (PBP3) in intact cells but inactivated (to various degrees) all PBPs except PBP5/6 in lysed cells, suggesting poor penetration (Table 2). At the rather high studied concentrations, cefoxitin, carbenicillin and ticarcillin showed extensive binding in intact and lysed cells. While piperacillin yielded extensive binding in lysed cells, it only significantly inactivated PBP3 (and PBP4) in intact cells. The BLI showed limited binding in intact bacteria and some PBP binding in lysed cells (Table 3).

**TABLE 1:** Susceptibility data and estimated rate of net influx and PBP access for 15 drugs in intact cells of P. aeruginosa PAO1

β-Lactam sub-class	Drug	MIC^*a*^ (mg/L)	Studied extracellular drug conc. in PBP binding assays (mg/L)	Rate_Influx/access,scaled_^*b*^ (SE%) (no. of drug molecules/min/(mg/L))	Relative Rate_Influx/access,scaled_ compared to that of IPM	Rate_Influx/access_ at 2 × MIC^*d*^ (no. of drug molecules/min)
Carbapenem	Imipenem	1	2	74.5 (29.0%)	1.00	149
Carbapenem	Doripenem	1	2	40.9 (50.7%)	1.82	81.8
Carbapenem	Meropenem	0.5	1	35.1 (48.1%)	2.12	35.1
DBO-BLI^*e*^	Avibactam	ND^*f*^	4	9.85^*c*^ (30.9%)	7.56	39.4^*c*^
DBO-BLI	Relebactam	ND	4	9.85^*c*^ (30.9%)	*7.*56^*c*^	39.4^*c*^
Cephalosporin	Ceftazidime	1	2	5.18 (35.2%)	14.4	10.4
Cephalosporin	Cefepime	1	2	1.66 (39.4%)	44.9	3.32
BLI^*g*^	Sulbactam	ND	4	1.49 (45.1%)	50.0	5.96^*d*^
Carbapenem	Ertapenem	4	8	1.04 (35.2%)	71.6	8.32
Penicillin	Piperacillin	4	8	0.301 (81.6%)	248	2.41
Monobactam	Aztreonam	4	8	0.299 (57.1%)	249	2.39
BLI	Tazobactam	ND	4	0.208 (65.4%)	358	0.832^*d*^
Penicillin	Carbenicillin	48	96	0.148 (44.5%)	503	14.2
Penicillin	Ticarcillin	24	48	0.126 (19.5%)	591	6.05
Cephalosporin	Cefoxitin	1,024	2,048	0.0731 (39.7%)	1,019	150

a Broth microdilution MICs were performed following CLSI guidelines (70).

b The Rate_Influx/access,scaled_ has units of number of molecules per min and per mg/L of the extracellular drug concentration. A high Rate_Influx/access,scaled_ indicates that a drug has a high rate of net influx and PBP access.

c The Rate_Influx/access,scaled_ of relebactam was difficult to estimate and thus eventually estimated as a shared parameter with avibactam. Thus, the estimate for relebactam carries additional uncertainty due to limited PBP binding by relebactam in intact bacteria.

d This column shows the rate of net influx and PBP access at 2 × the MIC of the studied β-lactams. For β-lactams, the Rate_Influx/access_ is the product of the Rate_Influx/access,scaled_ and the studied extracellular drug concentration (i.e., 2 × MIC). For the BLIs, the column shows the Rate_Influx/access_ at the studied fixed concentration of 4 mg/L, since the MICs of the BLIs were high.

e DBO-BLI: Diazabicyclooctane-type BLI.

f ND: MIC not determined for β-lactamase inhibitors.

g BLI: β-lactamase inhibitor.

**TABLE 2:** Average ± SD of unbound PBPs over the first 60 min in the intact and lysed cell PBP assay^*a*^

Cells	PBP	IPM (2 mg/L)	DOR (2 mg/L)	MEM (1 mg/L)	ETP (8 mg/L)	CAZ (2 mg/L)	FEP (2 mg/L)	FOX (2048 mg/L)	ATM (8 mg/L)
Intact	1a	**0.29 ± 0.029** ^*b*^	**0.67 ± 0.037** ^*c*^	**0.68 ± 0.067** ^*c*^	0.87 ± 0.081^*d*^	**0.61 ± 0.052** ^*c*^	**0.61 ± 0.066** ^*c*^	**0.24 ± 0.018** ^*c*^	1.02 ± 0.064^*d*^
	1b	**0.30 ± 0.034** ^*b*^	**0.33 ± 0.024** ^*b*^	**0.37 ± 0.040** ^*c*^	**0.79 ± 0.070** ^*c*^	**0.87 ± 0.041** ^*c*^	0.94 ± 0.077^*d*^	**0.38 ± 0.013** ^*c*^	0.94 ± 0.065^*d*^
	2	**0.38 ± 0.078** ^*b*^	**0.30 ± 0.022** ^*b*^	**0.31 ± 0.035** ^*b*^	**0.63 ± 0.028** ^*c*^	**0.85 ± 0.077** ^*b*^	0.96 ± 0.125^*d*^	**0.45 ± 0.055** ^*b*^	1.02 ± 0.087^*e*^
	3	**0.35 ± 0.102** ^*b*^	**0.38 ± 0.024** ^*b*^	**0.44 ± 0.116** ^*b*^	**0.47 ± 0.027** ^*b*^	**0.51 ± 0.023** ^*c*^	**0.60 ± 0.094** ^*c*^	**0.47 ± 0.058** ^*b*^	**0.39 ± 0.034** ^*b*^
	4	**0.44 ± 0.081** ^*b*^	**0.22 ± 0.025** ^*b*^	**0.38 ± 0.103** ^*b*^	**0.33 ± 0.069** ^*b*^	**0.80 ± 0.058** ^*c*^	**0.69 ± 0.078** ^*c*^	**0.27 ± 0.054** ^*b*^	1.01 ± 0.024^*d*^
	5	**0.27 ± 0.009** ^*c*^	**0.36 ± 0.058** ^*b*^	**0.56 ± 0.055** ^*b*^	0.93 ± 0.090^*d*^	**0.83 ± 0.057** ^*b*^	0.98 ± 0.055^*e*^	**0.18 ± 0.029** ^*b*^	0.97 ± 0.067^*e*^
Lysed	1a	**0.19 ± 0.015** ^*e*^	**0.26 ± 0.020** ^*e*^	**0.29 ± 0.007** ^*e*^	**0.31 ± 0.022** ^*e*^	**0.17 ± 0.005** ^*e*^	**0.15 ± 0.006** ^*e*^	**0.14 ± 0.006** ^*e*^	**0.26 ± 0.018** ^*e*^
	1b	**0.18 ± 0.003** ^*e*^	**0.26 ± 0.022** ^*e*^	**0.21 ± 0.004** ^*e*^	**0.27 ± 0.012** ^*e*^	**0.36 ± 0.102** ^*e*^	**0.34 ± 0.021** ^*e*^	**0.16 ± 0.014** ^*e*^	**0.24 ± 0.017** ^*e*^
	2	**0.37 ± 0.034** ^*e*^	**0.34 ± 0.104** ^*e*^	**0.37 ± 0.047** ^*e*^	**0.35 ± 0.016** ^*e*^	**0.57 ± 0.098** ^*e*^	**0.47 ± 0.046** ^*e*^	**0.37 ± 0.105** ^*e*^	**0.64 ± 0.128** ^*e*^
	3	**0.52 ± 0.027** ^*e*^	**0.47 ± 0.066** ^*e*^	**0.57 ± 0.025** ^*e*^	**0.35 ± 0.053** ^*e*^	**0.21 ± 0.060** ^*e*^	**0.14 ± 0.005** ^*e*^	**0.43 ± 0.039** ^*e*^	**0.23 ± 0.059** ^*e*^
	4	**0.21 ± 0.0001** ^*e*^	**0.23 ± 0.014** ^*e*^	**0.24 ± 0.011** ^*e*^	**0.20 ± 0.005** ^*e*^	**0.35 ± 0.079** ^*e*^	**0.17 ± 0.020** ^*e*^	**0.19 ± 0.039** ^*e*^	**0.36 ± 0.055** ^*e*^
	5	**0.19 ± 0.010** ^*e*^	**0.43 ± 0.035** ^*e*^	**0.54 ± 0.046** ^*e*^	**0.40 ± 0.011** ^*e*^	**0.82 ± 0.066** ^*e*^	1.02 ± 0.042^*e*^	**0.13 ± 0.002** ^*e*^	1.01 ± 0.051^*e*^

a The extent of PBP binding was calculated via non-compartmental analysis using the linear trapezoidal rule to obtain the AUC_0-60min_ data. A number of 1.00 indicates no PBP binding, whereas 0.00 would indicate complete PBP inactivation. Bold numbers indicate statistically significant binding (compared to 1.00).

b Dark gray shading means no statistically significant difference between PBP inactivation in intact versus lysed cells for the respective PBP.

c Medium gray shading means significantly less PBP binding in intact versus lysed cells for the respective PBP.

d Light gray shading means no statistically significant extent of binding (compared to 1.00) in intact cells, but statistically significant binding in lysed cells, and statistically significant more PBP inactivation in lysed versus intact cells.

e No shading indicates a lack of significant PBP binding (compared to 1.00) both for lysed and intact cells.

**TABLE 3:** Average ± SD of unbound PBPs over the first 60 min in the intact and lysed cell PBP assay^*a*^

Cells	PBP	PIP (8 mg/L)	CAR (96 mg/L)	TIC (48 mg/L)	AVI (4 mg/L)^*b*^	REL (4 mg/L)^*b*^	SUL (4 mg/L)	TZB (4 mg/L)^*b*^
Intact	1a	0.79 ± 0.180^*e*^	**0.41 ± 0.006** ^*d*^	**0.48 ± 0.013** ^*d*^	0.93 ± 0.108^*f*^	**0.87 ± 0.067** ^*f*^	0.88 ± 0.167^*f*^	0.98 ± 0.207^*f*^
	1b	0.89 ± 0.238^*e*^	**0.36 ± 0.004** ^*d*^	**0.43 ± 0.007** ^*d*^	0.78 ± 0.116^*f*^	**0.84 ± 0.076** ^*f*^	**0.75 ± 0.116** ^*f*^	1.05 ± 0.186^*f*^
	2	0.91 ± 0.323^*f*^	**0.82 ± 0.018** ^*d*^	**0.77 ± 0.076** ^*d*^	0.80 ± 0.131^*f*^	**0.81 ± 0.076** ^*f*^	0.97 ± 0.180^*f*^	1.04 ± 0.157^*f*^
	3	**0.48 ± 0.023** ^*d*^	**0.39 ± 0.088** ^*c*^	**0.48 ± 0.030** ^*d*^	0.89 ± 0.122^*f*^	**0.81 ± 0.070** ^*f*^	0.98 ± 0.143^*f*^	1.10 ± 0.258^*f*^
	4	**0.69 ± 0.031** ^*d*^	**0.49 ± 0.141** ^*c*^	**0.59 ± 0.036** ^*d*^	**0.75 ± 0.118** ^*c*^	**0.84 ± 0.053** ^*f*^	0.74 ± 0.208^*f*^	1.06 ± 0.292^*f*^
	5	1.11 ± 0.105^*f*^	**0.80 ± 0.033** ^*d*^	1.08 ± 0.075^*e*^	0.90 ± 0.088^*e*^	**0.90 ± 0.023** ^*f*^	0.84 ± 0.102^*f*^	1.14 ± 0.185^*f*^
Lysed	1a	**0.14 ± 0.004** ^*f*^	**0.13 ± 0.002** ^*f*^	**0.10 ± 0.048** ^*f*^	1.07 ± 0.101^*f*^	0.95 ± 0.077^*f*^	**0.68 ± 0.025** ^*f*^	**0.79 ± 0.033** ^*f*^
	1b	**0.14 ± 0.005** ^*f*^	**0.15 ± 0.021** ^*f*^	**0.14 ± 0.007** ^*f*^	**0.64 ± 0.034** ^*f*^	0.98 ± 0.075^*f*^	**0.48 ± 0.011** ^*f*^	**0.73 ± 0.023** ^*f*^
	2	**0.18 ± 0.032** ^*f*^	**0.20 ± 0.018** ^*f*^	**0.32 ± 0.055** ^*f*^	**0.79 ± 0.100** ^*f*^	0.88 ± 0.087^*f*^	0.79 ± 0.138^*f*^	1.10 ± 0.032^*f*^
	3	**0.23 ± 0.018** ^*f*^	**0.17 ± 0.022** ^*f*^	**0.17 ± 0.014** ^*f*^	1.07 ± 0.041^*f*^	1.05 ± 0.065^*f*^	1.09 ± 0.013^*f*^	1.05 ± 0.015^*f*^
	4	**0.14 ± 0.007** ^*f*^	**0.17 ± 0.033** ^*f*^	**0.15 ± 0.008** ^*f*^	**0.61 ± 0.069** ^*f*^	**0.82 ± 0.077** ^*f*^	**0.70 ± 0.061** ^*f*^	0.97 ± 0.025^*f*^
	5	0.82 ± 0.091^*f*^	**0.58 ± 0.052** ^*f*^	**0.67 ± 0.091** ^*f*^	**0.49 ± 0.020** ^*f*^	**0.86 ± 0.053** ^*f*^	**0.46 ± 0.008** ^*f*^	0.90 ± 0.056^*f*^

a The extent of PBP binding was calculated via non-compartmental analysis using the linear trapezoidal rule to obtain the AUC_0-60min_ data. A number of 1.00 indicates no PBP binding, whereas 0.00 would indicate complete PBP inactivation. Bold number indicated statistically significant binding (compared to 1.00).

b Fixed beta-lactamase inhibitor concentration of 4 mg/L (i.e., concentration is lower than 2 × MIC).

c Dark gray shading means no statistically significant difference between PBP inactivation in intact versus lysed cells for the respective PBP.

d Medium gray shading means significantly less PBP binding in intact versus lysed cells for the respective PBP.

e Light gray shading means no statistically significant extent of binding (compared to 1.00) in intact cells, but statistically significant binding in lysed cells, and statistically significant more PBP inactivation in lysed versus intact cells.

f No shading indicates a lack of significant PBP binding (compared to 1.00) both for lysed and intact cells.

### QSP modeling.

When we simultaneously modeled the time course of PBP binding in intact and lysed bacteria, imipenem displayed rapid and extensive penetration and binding of all PBPs in intact and lysed cells (Fig. 3), with rapid binding of PBP1a, 1b, 2, and 3. This was also shown by the estimated second-order acylation rate constants (Table S1). While cefoxitin also penetrated and bound PBPs rapidly, it required a much higher concentration (2,048 mg/L) than imipenem (2 mg/L). In contrast, PBP binding by meropenem and doripenem was slower in intact compared to that in lysed cells and limited for PBP5/6 (Fig. 3 and Fig. S7). Ertapenem displayed slower binding of PBP1a and 1b in lysed cells, and much slower overall binding in intact compared to that in lysed cells, demonstrating slow penetration. Aztreonam, cefepime, ceftazidime, piperacillin, carbenicillin, and ticarcillin achieved reasonably rapid PBP binding in lysed cells. However, PBP binding in intact cells was very slow for aztreonam and piperacillin. In intact cells, cefepime, ceftazidime, carbenicillin, and ticarcillin mostly inactivated PBP1a and 3; carbenicillin and ticarcillin additionally bound PBP1b (and PBP4). The BLIs showed slow inactivation of some PBPs in lysed cells, but only avibactam and to a lesser extent sulbactam and relebactam displayed noticeable PBP binding in intact bacteria (Fig. 3).

The rate of net influx and PBP access (Rate_Influx/access_) represents the number of β-lactam molecules that pass the outer membrane and bind to PBPs per time at the studied extracellular drug concentration. We estimated the scaled net influx rate (Rate_Influx/access,scaled_) which represents the Rate_Influx/access_ divided by the extracellular drug concentration (Fig. S6). A high Rate_Influx/access,scaled_ indicated that a drug had a faster rate of net influx and PBP access. The Rate_Influx/access,scaled_ was fastest for imipenem, and approximately 2-fold slower for doripenem and meropenem. In contrast, ertapenem was 71.6-fold slower than imipenem indicating poor penetration (Table 1). Ceftazidime penetrated approximately 3-fold faster than cefepime. Compared to imipenem, the Rate_Influx/access,scaled_ was ~ 249-fold slower for piperacillin and aztreonam, 503-fold slower for carbenicilin, 591-fold slower for ticarcillin, and 1,019-fold slower for cefoxitin. Relative to imipenem, the Rate_Influx/access,scaled_ was 7.56-fold slower for avibactam, 50.0-fold slower for sulbactam, and 358-fold slower for tazobactam. We used the estimate from avibactam to model relebactam, since PBP binding by relebactam in intact cells was limited. Thus, the Rate_Influx/access,scaled_ estimate for relebactam is associated with a larger uncertainty.

### PBP5/6 binding - sink target.

**FIG 4:**
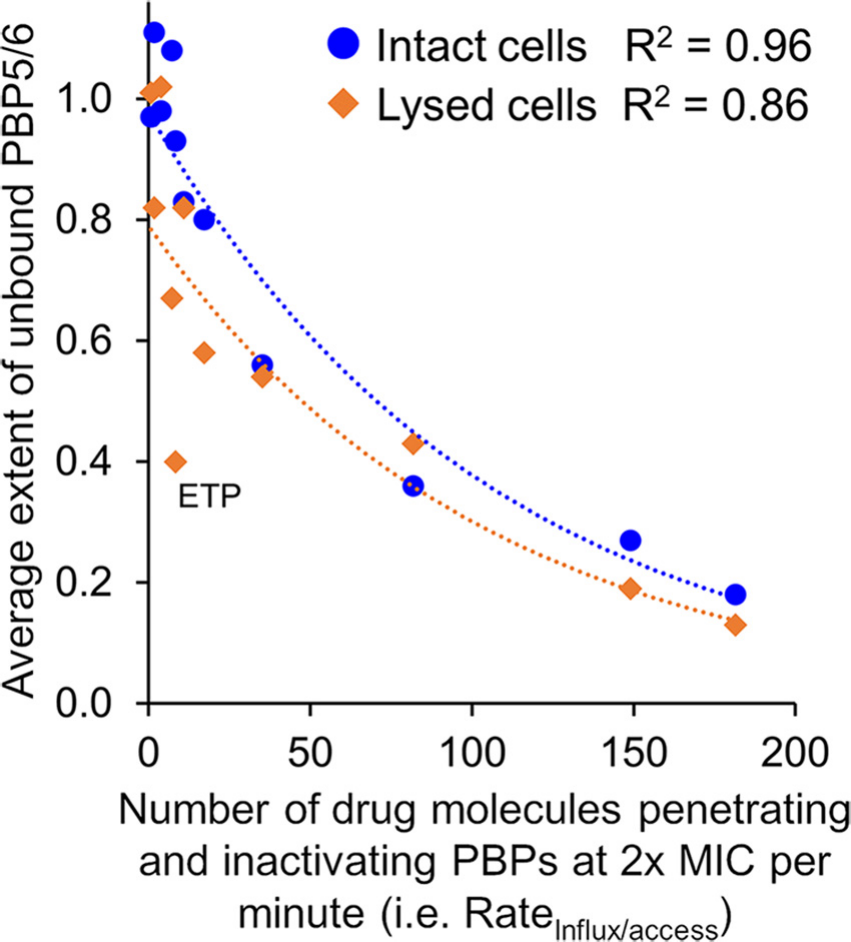
Empirical relationship between the extent of PBP5/6 binding and the number of β-lactam molecules that penetrate the outer membrane and inactivate PBPs per minute at 2 × the MIC (i.e., Rate_Influx/access_). Please note that the Rate_Influx/access_ accounts for the extracellular drug concentration (as shown in the last column of Table 1). The plot only shows β-lactams, since the BLI were studied at 4 mg/L (and not at 2 × the MIC). The outlier at the bottom left for the lysed cell assay was ertapenem (ETP), which penetrated slowly. The plot highlights that β-lactams which inactivate the highly expressed PBP5/6 need to be rapidly penetrating (i.e., have a high rate of net influx and PBP access) to achieve 2 × MIC. In contrast, slowly penetration β-lactams can be efficacious (i.e., 2 × MIC), as long as they have limited or no PBP5/6 binding.

The estimated rate of net influx and PBP access (Rate_Influx/access_) at 2 × the β-lactam MIC showed a strong inverse correlation with the extent of PBP5/6 binding (i.e., AUC_0-60min_) in lysed and intact cells (Fig. 4). Ertapenem was an outlier in the lysed cell assay, since only the intact but not the lysed cell assay accounts for the poor penetration of ertapenem (Fig. 3). The β-lactams that inactivated PBP5/6 had to rapidly penetrate the outer membrane (i.e., have a high rate of net influx and PBP access) in order to achieve 2 × the MIC. In contrast, slowly penetrating β-lactams like aztreonam could achieve 2 × the MIC, as long as they did not or only minimally bound PBP5/6.

## DISCUSSION

The PBPs are among the most valuable drug targets ever discovered, resulting in β-lactams being the most widely used class of antibiotics (33, 34). All bacteria express multiple different PBPs, which are the primary targets of all β-lactams. These drugs covalently bind to and thereby inactivate 1 or multiple PBPs. However, the expression of PBPs differs substantially. This offers a unique opportunity to study whole-cell receptor binding through covalent PBP binding, while accounting for the relative abundance of each PBP. This mass balance situation in the presence of the OM as a penetration barrier is neither captured by PBP binding studies using isolated membranes from lysed bacteria nor by the fluorescence anisotropy assay which assessed individual PBPs (19). The latter types of studies provide valuable mechanistic insights and thus complement whole-cell PBP binding data.

By comparing the rate and extent of PBP binding in intact and lysed cells for 15 drugs in P. aeruginosa, we found that carbapenems (except ertapenem) had the fastest rate of net influx and PBP access, followed by avibactam, ceftazidime, and cefepime (Table 1). The penicillin and aztreonam had considerably slower rates than imipenem. We identified, for the first time, a quantitative relationship between PBP5/6 binding and the rate of net influx and PBP access at 2 × the MIC of β-lactams (Fig. 4). With PBP5/6 as dominantly expressed PBP in P. aeruginosa, we identified PBP5/6 as a decoy target (i.e., a sink) which precludes β-lactams from inactivating the more essential PBPs (i.e., PBPs 1a, 1b, 2, and 3). This study was not designed to identify the relative importance of these PBPs toward bacterial killing.

With the global spread of resistant isolates of P. aeruginosa and multidrug-resistant high-risk clones continuously evolving to become resistant to the most recently approved antibiotics, few efficacious treatment options remain (35–38). The β-lactams provide excellent efficacy and safety and have been a cornerstone of antimicrobial therapy against susceptible P. aeruginosa isolates (32). To combat multidrug-resistant isolates, combination therapies of β-lactams with novel BLI, polymyxins, aminoglycosides, or quinolones have been studied (39–41). However, these combinations were associated with considerable toxicity, as well as suboptimal clinical and microbiological outcomes and emergence of resistance, especially for serious infections (42–44).

To enhance the clinical utility of available β-lactams, 2 main strategies have been pursued. First, combining β-lactams with other antibiotic classes that enhance the OM permeability and target site penetration (45, 46), and second using a BLI which protects the β-lactam from β-lactamase-related hydrolysis. Both of these strategies enhance the rate of net influx and PBP access via the partner drug. In some cases, the BLI may contribute some PBP binding in addition to the β-lactamase inhibitory effect (13, 14, 47–49). For example, novel diazabicyclooctane (DBO) BLIs bind PBP2 in P. aeruginosa, as we recently studied for avibactam in intact and lysed cells (50). To quantify the periplasmic target site penetration, we developed a novel assay and systematically assessed the time course and extent of covalent PBP binding for 15 β-lactams and BLIs in P. aeruginosa.

This study confirmed that PBP5/6 was the most abundant PBP in P. aeruginosa (16), with an at least ~ 8-fold higher expression than that of any other PBPs (Fig. 1). This offers the possibility of PBP5/6 acting as a decoy target, where β-lactam molecules binding to PBP5/6 are no longer available to reach their essential targets (i.e., PBP1a, 1b, 2, and 3) (51). While P. aeruginosa PBP5 mainly functions as a DD-carboxypeptidase, this highly expressed PBP has been shown to have some β-lactamase activity to primarily hydrolyze penicillins and carbapenems (e.g., meropenem and doripenem) (51, 52). This feature highlights the role of PBP5/6 as a highly expressed decoy target with additional β-lactamase activity, which may serve as a component of β-lactam resistance in strains with limited activity of other β-lactamase(s) and of efflux pump(s).

The expression of PBP2 and PBP3 was low compared to that of PBP5/6 and slightly increased over 60 min (Fig. 1) due to growth-phase related differential expression, in agreement with prior reports (52, 53). The PBP3 is known to have allosteric regulation-dependent catalytic activity, requiring previous engagement with other divisome proteins for its activation (54, 55). Furthermore, OM protease-related hydrolysis (*ompT*) can alter PBP3 integrity (17, 21). Taken together, the expression differs substantially between various PBPs and changes with growth phase. This makes PBPs moving targets, which is likely an important feature for optimizing β-lactam therapies, since PBP2 and PBP3 represent critical targets to kill P. aeruginosa rapidly and extensively during different growth phases (56, 57).

It has been previously observed that the deacylation half-life is less clinically relevant for PBPs, since it is slower than the rate of bacterial replication (58, 59). In agreement with these prior reports, we found no significant dissociation over 60 min for any of the drugs tested. All carbapenems showed extensive PBP binding in lysed cells (Fig. 3), in agreement with published lysed cell IC_50_ data (20, 60, 61). However, in intact cells, imipenem showed the most rapid and extensive PBP inactivation, followed by doripenem and meropenem, and ertapenem yielding the least PBP binding (Fig. 3, and Tables 1 and 2). This rapid penetration and PBP binding by imipenem explained why only imipenem achieved ~ 1.5 log_10_ killing by 1 h (Fig. S1), despite all 4 carbapenems having similar PBP IC_50_s in P. aeruginosa when using the isolated membrane assay (62). The present study also showed that the poor OM penetration of ertapenem significantly contributed to its lack of clinically relevant activity against P. aeruginosa (63).

Cefoxitin achieved considerable PBP binding in intact bacteria (Table 2). However, this required a very high extracellular concentration, resulting in a slow scaled rate of net influx and PBP access (Table 1). This estimated slow rate for cefoxitin is likely affected (i.e., biased low) by the rapid induction of the AmpC β-lactamase (within ~ 20 min at the protein level) due to PBP4 binding by cefoxitin, and by cefoxitin being a substrate of this cephalosporinase (6, 64). Overall, this explains a lack of cefoxitin activity in P. aeruginosa. Similarly for penicillins, the scaled rate of net influx and PBP access was relatively slow for piperacillin, and even slower for carbenicillin and ticarcillin (both double anions) (Tables 1 and 3). These small estimates are likely in part caused by the penicillins being inactivated by AmpC or effluxed by MexAB-OprM (65).

Binding of PBPs by ceftazidime and cefepime was markedly less in intact compared to that in lysed cells. The rate of net influx and PBP access was ~ 3-times faster for ceftazidime than that of cefepime, potentially due to efflux of cefepime (and cefoxitin) by MexAB-OprM and MexXY-OprM (66), and due to limited efflux of ceftazidime by MexAB-OprM (67). As a feature of our whole cell target site penetration approach, this assay quantifies the covalent binding of PBPs and thus captures only the drug molecules that ultimately bind their PBP targets. It is a major strength of this approach that the estimated rate of net influx and PBP access accounts for the impact of all expressed resistance mechanisms (e.g., efflux, β-lactamases, or loss of an OM porin).

In general, the lysed cell PBP IC_50_s (20, 60, 61) predicted the primary PBP targets in the intact cell assay (Fig. 3). As expected, for slowly penetrating β-lactams, the extent of PBP binding was diminished in intact compared to that in lysed cells for all PBPs or at least for the non-primary target PBPs (Tables 2 and 3). While aztreonam bound several PBPs in lysed cells, it only significantly inactivated its primary target PBP3 in intact cells, in agreement with aztreonam lysed cell IC_50_ data (60, 61) and a prior report on whole-cell binding of aztreonam in E. coli (12). Future studies are warranted to further investigate potential allosteric interactions among the proteins that are part of the elongasome and divisome complexes, potential conformational changes in periplasm, and the inner membrane architecture related to PBP target accessibility (19).

To combat P. aeruginosa with specific resistance mechanisms, the large number of clinically available β-lactams allows one to combine 2 β-lactams which are not affected by the same resistance determinants. Furthermore, novel BLIs have been extensively used to protect their partner β-lactams (13, 14, 47–49). While approaches to avoid or inhibit resistance mechanisms are clearly beneficial, fundamental insights on the target site penetration of β-lactams and their interplay with binding of different PBPs provide important insights (50). Of note, PBP expression can change considerably with growth phase (53). The novel assay developed here can account for β-lactamase-related hydrolysis, efflux, differential expression of OM porins, and drug-drug interactions, since all these mechanisms decrease the observed covalent PBP binding. However, the latter is directly related to bacterial killing by β-lactams. Future studies on isogenic strains with knockout or over-expression of specific efflux pumps, β-lactamases, and OM porins are clearly warranted to further enhance the insights from this novel assay, but were beyond the scope of the present study. This would further expand and ‘validate’ the applicability of this novel whole cell PBP binding assay. Overall, this approach brings our understanding of the mechanisms of β-lactams action and their target site penetration in intact bacteria to the next level (19).

The proposed assay can be readily expanded to studying PBP binding by double β-lactams combinations with or without a BLI in resistant strains, while using flow cytometry and confocal microscopy to confirm morphology changes due to PBP binding (19). Moreover, future studies should assess the rate and extent of PBP binding in different growth phases and physiological states (e.g., stationary phase, slow and non-replicating persisters, and bacteria in biofilms). This would provide mechanistic insights for further optimizing β-lactam combination therapies via complementary PBP binding, β-lactamase inhibition, and enhancing target site penetration. These mechanisms can be readily implemented into the developed QSP model for PBP binding. This approach can predict the extent and time course of PBP inactivation using human plasma concentration time profiles and between patient variability in pharmacokinetics for monotherapies and combinations.

A limitation of our study is the use of a single P. aeruginosa strain and lack of studies on isogenic strains with knockout or over-expression of efflux, β-lactamases, and OM porins. However, our comprehensive data set comprised 15 drugs studied at least in duplicate. We used a single drug concentration and therefore had to fix the Km. Informed by mechanistic prior studies (58), we modeled PBP binding in periplasm to be linear (i.e., non-saturable) using second-order acylation rate constants. Some of the latter were fixed (after in-depth sensitivity analyses) for the highest affinity targets due to our first sample being at 15 min (Table S1). We observed slightly more noise in the lysed but not in the intact cell assay for imipenem and meropenem (but not for the other carbapenems) for PBP3. Our results for carbapenems will likely change with altered OM porin expression (e.g., loss of OprD). Moreover, isolate-to-isolate genomic variability between the different laboratory-adapted reference strains in various laboratories worldwide may affect the results. It is well known that PAO1 strains present genomic and phenotypic diversity that may affect target site penetration. However, limited changes in β-lactam susceptibility have been observed for any of the tested antimicrobial agents in any of the in different PAO1 lineages studied (68, 69). Furthermore, the MIC changes were not correlated with small nuclear polymorphisms (SNP) detected in genes that take part in OM permeability regulation (e.g., non-synonymous SNPs in *mexT*; which activates the expression of the MexEF-OprN efflux pump and represses OprD expression) (68, 69). A potential further limitation is studying a relatively short duration (60 min). While this allowed us to systematically compare the OM penetration and PBP binding between different drugs, a longer duration may have provided additional insights for slowly penetrating drugs. However, such studies would likely be subject to more pronounced growth phase related PBP expression changes.

In summary, this study identified PBP5/6 to be an important sink target and showed that β-lactams which bind PBP5/6 had to have a rapid rate of net influx and PBP access in order to achieve 2 × the MIC. Conversely, slowly penetrating β-lactams could achieve 2 × the MIC, if they had limited or no PBP5/6 binding. We comprehensively compared the rate and extent of PBP binding in intact and lysed P. aeruginosa and found that carbapenems (except ertapenem) rapidly inactivated PBPs, followed by cephalosporins (except cefoxitin), penicillins and aztreonam. Avibactam penetrated relatively fast. The proposed assay characterizes the rate of net influx and PBP access of drug molecules which ultimately covalently bind to PBPs in the presence of all expressed resistance mechanisms. Thus, the assay captures the pharmacologically active drug molecules (i.e., those that inactivate PBPs), which is arguably the most important predictor for bacterial killing. These novel mechanistic insights enhance our understanding of the rate and extent of PBP binding in intact bacteria and provide tangible guidance for optimizing future PBP-binders.

## MATERIALS AND METHODS

### Antibiotics *in vitro* susceptibility testing.

β-Lactam antibiotics used were ertapenem (Merck Sharp & Dohme), imipenem (Fresenius Kabi), meropenem (Aurovitas), cefepime (Accord Healthcare), cefoxitin and ceftazidime from Laboratorios Normon (), aztreonam (Bristol-Myers Squibb), as well as doripenem, carbenicillin, piperacillin, ticarcillin, avibactam, relebactam, sulbactam, and tazobactam from MedChem Express. The β-lactam MICs were determined at least in duplicate in strain P. aeruginosa PAO1 following the Clinical and Laboratory Standards Institute (CLSI) broth microdilution method (70).

### Whole cell PBP binding assay.

We determined the extent of PBP binding in intact cells of PAO1 at 0, 15, 30, and 60 min after incubation with 10 structurally different β-lactams (4 carbapenems, 2 cephalosporins, 3 penicillin, and 1 monobactam) and 3 BLI. Late exponential growth-phase P. aeruginosa PAO1 cultures (7.6 log_10_ CFU/mL) were incubated in cation-adjusted Mueller-Hinton broth (CAMHB; Oxoid) at 37°C (180 rpm) containing 2 × MIC (Table 1) of each β-lactam. Avibactam, relebactam, sulbactam, and tazobactam were studied at a fixed concentration of 4 mg/L that is within the clinically relevant range for these BLIs. A volume of 50 mL from control and treatment samples were taken 15, 30, and 60 min post-incubation, kept on ice, and centrifuged (3,220 × *g* for 10 min at 4°C). Bacterial pellets were washed in 30 mL of phosphate-buffered saline (PBS) buffer (pH 7.5) four times, resuspended in 10 mL of PBS, and gently sonicated using a Digital Sonifier Unit model S-450D (Branson Ultrasonics Corp.) immersed in an ice bath. Membranes containing antibiotic-bound PBPs were collected by ultracentrifugation (150,000 × *g*, 30 min, 4°C) using an Optima L100XP Ultra centrifuge (Beckman Coulter, Inc.) and resuspended in 60 μL of PBS. Total protein content was measured using the Quick Start Bradford protein assay kit with bovine serum albumin as standard (Bio-Rad Laboratories), according to the manufacturer’s instructions.

To determine the unbound fraction of PBPs, membranes containing PBPs (10 μg) were labeled with 25 μM the fluorescent penicillin probe, Boc-FL, for 15 min at 37°C (18). Adding Boc-FL at this high concentration labels all unbound PBPs. Labeled PBPs were separated through 4 to 15% SDS-polyacrylamide gels (Bio-Rad Laboratories) and visualized using a Typhoon FLA 9500 biomolecular imager (GE Healthcare Bio-Sciences AB) with an excitation wavelength of 488 nm and emission at 530 nm. Binding to different PBPs was determined from at least 3 independent experiments using the ImageQuant TL software (version v8.1.0.0, GE Healthcare Bio-Sciences AB).

Serial viable counts were determined to assess bacterial growth and killing up to 60 min. To rule out any significant dissociation of the covalent PBP-drug complex or displacement of the drug by Boc-FL, we carefully optimized the incubation times and labeling conditions for the intact cell binding studies described above for selected compounds from each subgroup of β-lactams. We further optimized the amount of protein loaded on the gels as well as the Boc-FL concentration. To determine the PBP binding profiles without the penetration barrier imposed by the outer membrane, the same PBP binding experiments were carried out with isolated PBP-containing membranes following previously described protocols (14). Briefly, samples (of 50 mL each) were obtained from late exponential growth-phase P. aeruginosa PAO1 cultures (7.6 log_10_ CFU/mL) and incubated 15, 30, and 60 min at 37°C with the previously described compounds, and afterwards labeled with Boc-FL (25 μM). Determination of the unbound PBP fractions was performed as described above. This allowed us to compare the rate and extent of PBP binding between lysed and intact bacteria for each compound, and to thereby draw inferences about their rates of target site penetration and access to different PBPs.

### Statistical data analysis.

The GraphPad Prism software (version v5.01) was used for graphical representation and statistics. For each replicate, the fraction of unbound PBPs was calculated by normalizing the band intensity of each PBP band against the intensity of the respective PBP and replicate at 0 h (i.e., before adding drug). A value of 1.0 for the fraction of unbound PBPs represents no binding and a value of 0.0 complete PBP binding (i.e., inactivation). Data on PBP7 were not reported, since PBP7 had relatively low expression, its data were rather noisy, and PBP7 did not show a clear trend of binding.

The average extent of unbound PBPs (AUC_0-60min_) was calculated over the observed 60 min using the linear trapezoidal rule in the Phoenix 64 WinNonlin Professional software (version 8.3.4.295, Certara). Statistically significant binding was concluded if the extent of PBP binding differed significantly from 1.0. Subsequently, we used linear mixed-effects modeling to compare the least square means of the AUC_0-60min_ for each PBP between lysed and intact cells of each drug.

### Quantitative and systems pharmacology modeling.

The individual PBP binding profiles were modeled for each PBP, replicate, and drug via population modeling. We compared the overall kinetics of PBP binding for the 6 receptors simultaneously and did not seek to resolve the micro-constants of the binding reactions.

### Mass balance equations.

To implement mass balance, we accounted for the relative band intensity across PBP1a, PBP1b, PBP2, PBP3, PBP4, and PBP5/6. We are not aware of published data on the total number of PBP molecules per cell in P. aeruginosa. Therefore, we compared the PBP expression between E. coli and P. aeruginosa (Tab. S3) and then borrowed the published number of PBPs (i.e., 1,731) in rich broth medium from E. coli (16). Based on this total number of PBP molecules per cell, the relative band intensities from P. aeruginosa were used to split these among the 6 PBPs. These were 153 molecules for PBP1a (equivalent to 8.8% of all PBPs), 118 (6.8%) for PBP1b, 50 (2.9%) for PBP2, 79 (4.6%) for PBP3, 99 (5.7%) for PBP4, and 1,232 (71.2%) for PBP5/6. We estimated a random variable (e.g., Fini_1a_) for a potential small deviation of the initial condition from the nominal value of PBP molecules per cell at 0 h for each dependent variable (e.g., 153 for PBP1a) (Fig. S8).

### Simultaneous binding of 6 PBPs.

To describe the simultaneous binding of 6 PBPs, we implemented 7 coupled differential equations, i.e., 1 for each PBP and the number of drug molecules available for PBP binding at the target site. For studies in lysed bacteria, this number of drug molecules was initialized at a larger number (10,000,000) to reflect a vast excess compared to the number of PBPs (i.e., 1,731) and net influx rate was set to zero. In intact bacteria, the number of drug molecules at the periplasmic target site was initialized at zero and the rate of net influx and PBP access (Rate_Influx/access_) was estimated. The Rate_Influx/access_ represents the number of β-lactam molecules that penetrate periplasm and that bind one of the different PBPs (i.e., molecules that are neither effluxed nor inactivated by the AmpC β-lactamase). The Rate_Influx/access_ was scaled according to the extracellular β-lactam concentration (C_drug_) assuming that the penetration rate was linear (i.e., not saturable) (71).

(1) $$\mathrm{Rat}e_{\mathrm{Influx/access}}\;\mathrm{=Rat}e_{\mathrm{Influx/access,scaled}}\;\times \;C_{\mathrm{drug}}$$

The scaled net influx rate (Rate_Influx/access,scaled_) differs from a permeability coefficient, in that the former directly describes the number of drug molecules that penetrate and covalently bind to the 6 PBP target receptors. In contrast, when estimating a permeability coefficient, one would also need to account for drug loss due to β-lactamase activity, efflux, and passive diffusion out of periplasm. The number of drug molecules per cell in periplasm (N_Peri_) was then used in Michaelis-Menten equations to describe the binding to each PBP (as shown for PBP1a):

(2) $${\text{MM}}_{\text{1a}}=\frac{{\text{V}}_{\text{max,1a}}}{\text{Km}\;+\;{\text{N}}_{\text{Peri}}}$$

The V_max,1a_ is the maximum rate of acylation (unit: number of molecules per minute) for PBP1a and the Km is the Michaelis-Menten constant (unit: number of drug molecules). The Km was fixed to 1,000 drug molecules, because only one drug concentration was studied and thus the data set did not support estimation of Km. The chosen Km value assured linear (i.e., non-saturated) PBP binding in periplasm of the intact cell assay. In the lysed cell scenario, this means the PBP binding reaction was fully saturated in the presence of the large excess of drug molecules (i.e., 10^7^ = 10,000x Km). The Michaelis-Menten equations for the binding of the other PBPs used the same equation with separate maximum rates of acylation. The de-acylation rate was not modeled, as it is slow compared to the study duration of 60 min. This yields the following differential equation for the number of unbound PBP1a molecules per cell (N_PBP1a_):

(3) $$\frac{\text{d}\left({\text{N}}_{\text{PBP1a}}\right)}{\text{dt}}\;=\;-{\text{MM}}_{\text{1a}}\times {\text{N}}_{\text{Peri}}\;\times \;{\text{N}}_{\text{PBP1a}}$$

The N_Peri_ represents the number of drug molecules per cell in periplasm. The same equation was applied to all other PBPs. To reflect the competition in the access of a drug molecule in periplasm that can bind to one of the six PBPs, the differential equation for N_Peri_ contained the binding reaction terms for each of the six PBPs included in the model:

(4) $$\begin{array}{l} \frac{d\left(N_{\mathrm{Peri}}\right)}{\mathrm{dt}}\;\mathrm{=Rat}e_{\mathrm{Influx/access}}\;-\;MM_{\mathrm{1a}}\;\times \;N_{\mathrm{PBP1a}}\;-\;MM_{\mathrm{1b}}\;\times \;N_{\mathrm{PBP1b}}\;-\;MM_{2}\;\times \;N_{\mathrm{PBP2}}\;-\;MM_{3}\;\times \;N_{\mathrm{PBP3}}\;-\;MM_{4}\;\times \;N_{\mathrm{PBP4}}\;-\;MM_{\mathrm{5/6}}\;\times \;N_{\mathrm{PBP5/6}} \end{array}$$

Once a drug molecule bound to one of the six PBPs, the drug molecule was lost (i.e., ‘consumed’) and the number of unbound PBP molecules decreased accordingly.

## References

[1] Song SH, Li XX, Wan QQ, Ye QF. 2014. Risk factors for mortality in liver transplant recipients with ESKAPE infection. Transplant Proc 46:3560–3563. doi:10.1016/j.transproceed.2014.08.049. 25498089

[2] Sunenshine RH, Wright M-O, Maragakis LL, Harris AD, Song X, Hebden J, Cosgrove SE, Anderson A, Carnell J, Jernigan DB, Kleinbaum DG, Perl TM, Standiford HC, Srinivasan A. 2007. Multidrug-resistant Acinetobacter infection mortality rate and length of hospitalization. Emerg Infect Dis 13:97–103. doi:10.3201/eid1301.060716. 17370521 PMC2725827

[3] Kim YJ, Jun YH, Kim YR, Park KG, Park YJ, Kang JY, Kim SI. 2014. Risk factors for mortality in patients with *Pseudomonas aeruginosa* bacteremia; retrospective study of impact of combination antimicrobial therapy. BMC Infect Dis 14:161–167. doi:10.1186/1471-2334-14-161. 24655422 PMC3994322

[4] Spratt BG. 1975. Distinct penicillin binding proteins involved in the division, elongation, and shape of *Escherichia coli* K12. Proc Natl Acad Sci USA 72:2999–3003. doi:10.1073/pnas.72.8.2999. 1103132 PMC432906

[5] Sauvage E, Kerff F, Terrak M, Ayala JA, Charlier P. 2008. The penicillin-binding proteins: structure and role in peptidoglycan biosynthesis. FEMS Microbiol Rev 32:234–258. doi:10.1111/j.1574-6976.2008.00105.x. 18266856

[6] Moya B, Dötsch A, Juan C, Blázquez J, Zamorano L, Haussler S, Oliver A. 2009. Beta-lactam resistance response triggered by inactivation of a nonessential penicillin-binding protein. PLoS Pathog 5:e1000353. doi:10.1371/journal.ppat.1000353. 19325877 PMC2654508

[7] Lakaye B, Dubus A, Joris B, Frere JM. 2002. Method for estimation of low outer membrane permeability to beta-lactam antibiotics. Antimicrob Agents Chemother 46:2901–2907. doi:10.1128/AAC.46.9.2901-2907.2002. 12183245 PMC127435

[8] Drusano GL. 2011. What are the properties that make an antibiotic acceptable for therapy of community-acquired pneumonia? J Antimicrob Chemother 66 Suppl 3:61–67. doi:10.1093/jac/dkr100. 21482571

[9] Hancock RE, Woodruff WA. 1988. Roles of porin and beta-lactamase in beta-lactam resistance of *Pseudomonas aeruginosa*. Rev Infect Dis 10:770–775. doi:10.1093/clinids/10.4.770. 2460909

[10] Sato K, Nakae T. 1991. Outer membrane permeability of *Acinetobacter calcoaceticus* and its implication in antibiotic resistance. J Antimicrob Chemother 28:35–45. doi:10.1093/jac/28.1.35. 1722802

[11] Iyobe S, Watanabe M, Mitsuhashi S, Inoue M. 1999. Estimation of outer membrane permeability of carbapenem antibiotics to *Pseudomonas aeruginosa*. J Infect Chemother 5:168–170. doi:10.1007/s101560050028. 11810510

[12] Kocaoglu O, Carlson EE. 2015. Profiling of beta-lactam selectivity for penicillin-binding proteins in *Escherichia coli* strain DC2. Antimicrob Agents Chemother 59:2785–2790. doi:10.1128/AAC.04552-14. 25733506 PMC4394777

[13] Sutaria DS, Moya B, Green KB, Kim TH, Tao X, Jiao Y, Louie A, Drusano GL, Bulitta JB. 2018. First penicillin-binding protein occupancy patterns of beta-lactams and beta-lactamase inhibitors in *Klebsiella pneumoniae*. Antimicrob Agents Chemother 62:e00282-18. doi:10.1128/AAC.00282-18. 29712652 PMC5971569

[14] Moya B, Bhagwat S, Cabot G, Bou G, Patel M, Oliver A. 2020. Effective inhibition of PBPs by cefepime and zidebactam in the presence of VIM-1 drives potent bactericidal activity against MBL-expressing *Pseudomonas aeruginosa*. J Antimicrob Chemother 75:1474–1478. doi:10.1093/jac/dkaa036. 32083659

[15] Satta G, Cornaglia G, Mazzariol A, Golini G, Valisena S, Fontana R. 1995. Target for bacteriostatic and bactericidal activities of beta-lactam antibiotics against *Escherichia coli* resides in different penicillin-binding proteins. Antimicrob Agents Chemother 39:812–818. doi:10.1128/AAC.39.4.812. 7785976 PMC162634

[16] Dougherty TJ, Kennedy K, Kessler RE, Pucci MJ. 1996. Direct quantitation of the number of individual penicillin-binding proteins per cell in *Escherichia coli*. J Bacteriol 178:6110–6115. doi:10.1128/jb.178.21.6110-6115.1996. 8892807 PMC178478

[17] Chalut C, Charpentier X, Remy MH, Masson JM. 2001. Differential responses of *Escherichia coli* cells expressing cytoplasmic domain mutants of penicillin-binding protein 1b after impairment of penicillin-binding proteins 1a and 3. J Bacteriol 183:200–206. doi:10.1128/JB.183.1.200-206.2001. 11114917 PMC94866

[18] Zhao G, Meier TI, Kahl SD, Gee KR, Blaszczak LC. 1999. BOCILLIN FL, a sensitive and commercially available reagent for detection of penicillin-binding proteins. Antimicrob Agents Chemother 43:1124–1128. doi:10.1128/AAC.43.5.1124. 10223924 PMC89121

[19] Lang Y, Shah NR, Tao X, Reeve SM, Zhou J, Moya B, Sayed ARM, Dharuman S, Oyer JL, Copik AJ, Fleischer BA, Shin E, Werkman C, Basso KB, Deveson Lucas D, Sutaria DS, Mégroz M, Kim TH, Loudon-Hossler V, Wright A, Jimenez-Nieves RH, Wallace MJ, Cadet KC, Jiao Y, Boyce JD, LoVullo ED, Schweizer HP, Bonomo RA, Bharatham N, Tsuji BT, Landersdorfer CB, Norris MH, Soo Shin B, Louie A, Balasubramanian V, Lee RE, Drusano GL, Bulitta JB. 2021. Combating multidrug-resistant bacteria by integrating a novel target site penetration and receptor binding assay platform into translational modeling. Clin Pharmacol Ther 109:1000–1020. doi:10.1002/cpt.2205. 33576025 PMC10662281

[20] Rahme C, Butterfield JM, Nicasio AM, Lodise TP. 2014. Dual beta-lactam therapy for serious Gram-negative infections: is it time to revisit? Diagn Microbiol Infect Dis 80:239–259. doi:10.1016/j.diagmicrobio.2014.07.007. 25308565

[21] Henderson TA, Dombrosky PM, Young KD. 1994. Artifactual processing of penicillin-binding proteins 7 and 1b by the OmpT protease of *Escherichia coli*. J Bacteriol 176:256–259. doi:10.1128/jb.176.1.256-259.1994. 8282705 PMC205039

[22] Nakae T, Nakajima A, Ono T, Saito K, Yoneyama H. 1999. Resistance to beta-lactam antibiotics in *Pseudomonas aeruginosa* due to interplay between the MexAB-OprM efflux pump and beta-lactamase. Antimicrob Agents Chemother 43:1301–1303. doi:10.1128/AAC.43.5.1301. 10223959 PMC89266

[23] Nikaido H. 1985. Role of permeability barriers in resistance to beta-lactam antibiotics. Pharmacol Ther 27:197–231. doi:10.1016/0163-7258(85)90069-5. 2412244

[24] Godfrey AJ, Hatlelid L, Bryan LE. 1984. Correlation between lipopolysaccharide structure and permeability resistance in beta-lactam-resistant *Pseudomonas aeruginosa*. Antimicrob Agents Chemother 26:181–186. doi:10.1128/AAC.26.2.181. 6435513 PMC284115

[25] Godfrey AJ, Bryan LE. 1987. Penetration of beta-lactams through *Pseudomonas aeruginosa* porin channels. Antimicrob Agents Chemother 31:1216–1221. doi:10.1128/AAC.31.8.1216. 2443074 PMC174906

[26] Zimmermann W, Rosselet A. 1977. Function of the outer membrane of *Escherichia coli* as a permeability barrier to beta-lactam antibiotics. Antimicrob Agents Chemother 12:368–372. doi:10.1128/AAC.12.3.368. 334063 PMC429920

[27] Kim TH, Tao X, Moya B, Jiao Y, Basso KB, Zhou J, Lang Y, Sutaria DS, Zavascki AP, Barth AL, Reeve SM, Schweizer HP, Deveson Lucas D, Boyce JD, Bonomo RA, Lee RE, Shin BS, Louie A, Drusano GL, Bulitta JB. 2020. Novel cassette assay to quantify the outer membrane permeability of five β-lactams simultaneously in carbapenem-resistant *Klebsiella pneumoniae* and *Enterobacter cloacae*. mBio 11:e03189-19. doi:10.1128/mBio.03189-19. 32047131 PMC7018653

[28] Legaree BA, Daniels K, Weadge JT, Cockburn D, Clarke AJ. 2007. Function of penicillin-binding protein 2 in viability and morphology of *Pseudomonas aeruginosa*. J Antimicrob Chemother 59:411–424. doi:10.1093/jac/dkl536. 17289762

[29] Spratt BG. 1983. Penicillin-binding proteins and the future of beta-lactam antibiotics. The Seventh Fleming Lecture. J Gen Microbiol 129:1247–1260. doi:10.1099/00221287-129-5-1247. 6352855

[30] Neu HC. 1983. Penicillin-binding proteins and role of amdinocillin in causing bacterial cell death. Am J Med 75:9–20. doi:10.1016/0002-9343(83)90089-x. 6311012

[31] Siriyong T, Murray RM, Bidgood LE, Young SA, Wright F, Parcell BJ, Voravuthikunchai SP, Coote PJ. 2019. Dual β-lactam combination therapy for multi-drug resistant *Pseudomonas aeruginosa* infection: enhanced efficacy *in vivo* and comparison with monotherapies of penicillin-binding protein inhibition. Sci Rep 9:9098–9110. doi:10.1038/s41598-019-45550-z. 31235728 PMC6591303

[32] Jiao Y, Moya B, Chen M-J, Zavascki AP, Tsai H, Tao X, Sutaria DS, Louie A, Boyce JD, Deveson Lucas D, Kim TH, Tsuji BT, Bonomo RA, Drusano GL, Bulitta JB. 2019. Comparable efficacy and better safety of double beta-lactam combination therapy versus beta-lactam plus aminoglycoside in Gram-negatives: a meta-analysis of randomized, controlled trials. Antimicrob Agents Chemother 63:e00425-19. doi:10.1128/AAC.00425-19. 30988147 PMC6591590

[33] Silver LL. 2007. Multi-targeting by monotherapeutic antibacterials. Nat Rev Drug Discov 6:41–55. doi:10.1038/nrd2202. 17159922

[34] Silver LL. 2011. Challenges of antibacterial discovery. Clin Microbiol Rev 24:71–109. doi:10.1128/CMR.00030-10. 21233508 PMC3021209

[35] Gomis-Font MA, Cabot G, Sánchez-Diener I, Fraile-Ribot PA, Juan C, Moya B, Zamorano L, Oliver A. 2020. *In vitro* dynamics and mechanisms of resistance development to imipenem and imipenem/relebactam in *Pseudomonas aeruginosa*. J Antimicrob Chemother 75:2508–2515. doi:10.1093/jac/dkaa206. 32514525

[36] Fraile-Ribot PA, Cabot G, Mulet X, Periañez L, Martín-Pena ML, Juan C, Pérez JL, Oliver A. 2018. Mechanisms leading to *in vivo* ceftolozane/tazobactam resistance development during the treatment of infections caused by MDR *Pseudomonas aeruginosa*. J Antimicrob Chemother 73:658–663. doi:10.1093/jac/dkx424. 29149337

[37] Boucher HW, Talbot GH, Benjamin DK, Bradley J, Guidos RJ, Jones RN, Murray BE, Bonomo RA, Gilbert D, Infectious Diseases Society of America. 2013. 10 x '20 Progress–development of new drugs active against gram-negative bacilli: an update from the Infectious Diseases Society of America. Clin Infect Dis 56:1685–1694. doi:10.1093/cid/cit152. 23599308 PMC3707426

[38] Payne DJ, Gwynn MN, Holmes DJ, Pompliano DL. 2007. Drugs for bad bugs: confronting the challenges of antibacterial discovery. Nat Rev Drug Discov 6:29–40. doi:10.1038/nrd2201. 17159923

[39] Bergen PJ, Tsuji BT, Bulitta JB, Forrest A, Jacob J, Sidjabat HE, Paterson DL, Nation RL, Li J. 2011. Synergistic killing of multidrug-resistant *Pseudomonas aeruginosa* at multiple inocula by colistin combined with doripenem in an *in vitro* pharmacokinetic/pharmacodynamic model. Antimicrob Agents Chemother 55:5685–5695. doi:10.1128/AAC.05298-11. 21911563 PMC3232764

[40] Flamm RK, Nichols WW, Sader HS, Farrell DJ, Jones RN. 2016. *In vitro* activity of ceftazidime/avibactam against Gram-negative pathogens isolated from pneumonia in hospitalised patients, including ventilated patients. Int J Antimicrob Agents 47:235–242. doi:10.1016/j.ijantimicag.2016.01.004. 26920105

[41] Livermore DM, Warner M, Mushtaq S, Woodford N. 2016. Interactions of OP0595, a novel triple-action Diazabicyclooctane, with beta-lactams against OP0595-resistant Enterobacteriaceae mutants. Antimicrob Agents Chemother 60:554–560. doi:10.1128/AAC.02184-15. 26552987 PMC4704216

[42] Bliziotis IA, Samonis G, Vardakas KZ, Chrysanthopoulou S, Falagas ME. 2005. Effect of aminoglycoside and beta-lactam combination therapy versus beta-lactam monotherapy on the emergence of antimicrobial resistance: a meta-analysis of randomized, controlled trials. Clin Infect Dis 41:149–158. doi:10.1086/430912. 15983909

[43] Vardakas KZ, Tansarli GS, Bliziotis IA, Falagas ME. 2013. beta-lactam plus aminoglycoside or fluoroquinolone combination versus beta-lactam monotherapy for *Pseudomonas aeruginosa* infections: a meta-analysis. Int J Antimicrob Agents 41:301–310. doi:10.1016/j.ijantimicag.2012.12.006. 23410791

[44] Lodise TP, Bassetti M, Ferrer R, Naas T, Niki Y, Paterson DL, Zeitlinger M, Echols R. 2022. All-cause mortality rates in adults with carbapenem-resistant Gram-negative bacterial infections: a comprehensive review of pathogen-focused, prospective, randomized, interventional clinical studies. Expert Rev Anti Infect Ther 20:707–719. doi:10.1080/14787210.2022.2020099. 34937518

[45] Yadav R, Bulitta JB, Wang J, Nation RL, Landersdorfer CB. 2017. Evaluation of pharmacokinetic/pharmacodynamic model-based optimized combination regimens against multidrug-resistant *Pseudomonas aeruginosa* in a murine thigh infection model by using humanized dosing schemes. Antimicrob Agents Chemother 61:e01268-17. doi:10.1128/AAC.01268-17. PMC5700304 28993331

[46] Bergen PJ, Bulman ZP, Saju S, Bulitta JB, Landersdorfer C, Forrest A, Li J, Nation RL, Tsuji BT. 2015. Polymyxin combinations: pharmacokinetics and pharmacodynamics for rationale use. Pharmacotherapy 35:34–42. doi:10.1002/phar.1537. 25630411 PMC5215892

[47] Moya B, Barcelo IM, Bhagwat S, Patel M, Bou G, Papp-Wallace KM, Bonomo RA, Oliver A. 2017. WCK 5107 (Zidebactam) and WCK 5153 are novel inhibitors of PBP2 showing potent “beta-lactam enhancer” activity against *Pseudomonas aeruginosa*, including multidrug-resistant metallo-beta-lactamase-producing high-risk clones. Antimicrob Agents Chemother 61:e02529-16. doi:10.1128/AAC.02529-16. 28289035 PMC5444176

[48] Li H, Estabrook M, Jacoby GA, Nichols WW, Testa RT, Bush K. 2015. *In vitro* susceptibility of characterized β-lactamase-producing strains tested with avibactam combinations. Antimicrob Agents Chemother 59:1789–1793. doi:10.1128/AAC.04191-14. 25534728 PMC4325778

[49] Lomovskaya O, Sun D, Rubio-Aparicio D, Nelson K, Tsivkovski R, Griffith DC, Dudley MN. 2017. Vaborbactam: spectrum of beta-lactamase inhibition and impact of resistance mechanisms on activity in Enterobacteriaceae. Antimicrob Agents Chemother 61:e01443-17. doi:10.1128/AAC.01443-17. 28848018 PMC5655098

[50] López-Argüello S, Montaner M, Oliver A, Moya B. 2021. Molecular basis of AmpC β-lactamase induction by Avibactam in *Pseudomonas aeruginosa*: PBP occupancy, live cell binding dynamics and impact on resistant clinical isolates harboring PDC-X variants. Int J Mol Sci 22:3051–3061. doi:10.3390/ijms22063051. 33802668 PMC8002452

[51] Smith JD, Kumarasiri M, Zhang W, Hesek D, Lee M, Toth M, Vakulenko S, Fisher JF, Mobashery S, Chen Y. 2013. Structural analysis of the role of *Pseudomonas aeruginosa* penicillin-binding protein 5 in β-lactam resistance. Antimicrob Agents Chemother 57:3137–3146. doi:10.1128/AAC.00505-13. 23629710 PMC3697341

[52] Philippe N, Pelosi L, Lenski RE, Schneider D. 2009. Evolution of penicillin-binding protein 2 concentration and cell shape during a long-term experiment with *Escherichia coli*. J Bacteriol 191:909–921. doi:10.1128/JB.01419-08. 19047356 PMC2632098

[53] Liao X, Hancock RE. 1997. Identification of a penicillin-binding protein 3 homolog, PBP3x, in *Pseudomonas aeruginosa*: gene cloning and growth phase-dependent expression. J Bacteriol 179:1490–1496. doi:10.1128/jb.179.5.1490-1496.1997. 9045804 PMC178857

[54] Ricard M, Hirota Y. 1973. Process of cellular division in *Escherichia coli*: physiological study on thermosensitive mutants defective in cell division. J Bacteriol 116:314–322. doi:10.1128/jb.116.1.314-322.1973. 4583216 PMC246424

[55] Tormo A, Ayala JA, de Pedro MA, Aldea M, Vicente M. 1986. Interaction of FtsA and PBP3 proteins in the *Escherichia coli* septum. J Bacteriol 166:985–992. doi:10.1128/jb.166.3.985-992.1986. 3011758 PMC215222

[56] Chen W, Zhang YM, Davies C. 2017. Penicillin-binding protein 3 ss essential for growth of *Pseudomonas aeruginosa*. Antimicrob Agents Chemother 61:e01651-16. doi:10.1128/AAC.01651-16. 27821444 PMC5192123

[57] Moya B, Jiao Y, Qian Y, Tao X, Louie A, Bulitta J. 2017. Inactivation of penicillin-binding protein 2 is critical for killing *Pseudomonas aeruginosa* at high but not at low bacterial density. Abstract P0237. 27th ECCMID, Vienna, Austria.

[58] Shapiro AB, Gu RF, Gao N, Livchak S, Thresher J. 2013. Continuous fluorescence anisotropy-based assay of BOCILLIN FL penicillin reaction with penicillin binding protein 3. Anal Biochem 439:37–43. doi:10.1016/j.ab.2013.04.009. 23603065

[59] Livermore DM. 1987. Clinical significance of beta-lactamase induction and stable derepression in gram-negative rods. Eur J Clin Microbiol 6:439–445. doi:10.1007/BF02013107. 3311738

[60] Davies TA, Page MG, Shang W, Andrew T, Kania M, Bush K. 2007. Binding of ceftobiprole and comparators to the penicillin-binding proteins of *Escherichia coli*, *Pseudomonas aeruginosa*, *Staphylococcus aureus*, and *Streptococcus pneumoniae*. Antimicrob Agents Chemother 51:2621–2624. doi:10.1128/AAC.00029-07. 17470659 PMC1913263

[61] Davies TA, Shang W, Bush K, Flamm RK. 2008. Affinity of doripenem and comparators to penicillin-binding proteins in *Escherichia coli* and *Pseudomonas aeruginosa*. Antimicrob Agents Chemother 52:1510–1512. doi:10.1128/AAC.01529-07. 18250190 PMC2292531

[62] Qian Y, Moya B, Zhang L, Tao X, Kim T, Jiao Y, Sutaria D, Bulitta JB. 2018. Imipenem kills *Pseudomonas aeruginosa* (PA) substantially faster than meropenem and doripenem most likely due to more rapid outer-membrane (OM) penetration. Abstract P0262. 28th ECCMID, Madrid, Spain.

[63] Sousa D, Castelo-Corral L, Gutiérrez-Urbón J-M, Molina F, López-Calviño B, Bou G, Llinares P. 2013. Impact of ertapenem use on *Pseudomonas aeruginosa* and *Acinetobacter baumannii* imipenem susceptibility rates: collateral damage or positive effect on hospital ecology? J Antimicrob Chemother 68:1917–1925. doi:10.1093/jac/dkt091. 23557925

[64] Torrens G, Hernández SB, Ayala JA, Moya B, Juan C, Cava F, Oliver A. 2019. Regulation of AmpC-driven β-lactam resistance in *Pseudomonas aeruginosa*: different pathways, different signaling. mSystems 4:e00524-19. doi:10.1128/mSystems.00524-19. 31796566 PMC6890930

[65] Livermore DM. 1984. Penicillin-binding proteins, porins and outer-membrane permeability of carbenicillin-resistant and -susceptible strains of *Pseudomonas aeruginosa*. J Med Microbiol 18:261–270. doi:10.1099/00222615-18-2-261. 6092639

[66] Barceló I, Cabot G, Palwe S, Joshi P, Takalkar S, Periasamy H, Cortés-Lara S, Zamorano L, Sánchez-Diener I, Moya B, Bhagwat S, Patel M, Oliver A. 2021. *In vitro* evolution of cefepime/zidebactam (WCK 5222) resistance in *Pseudomonas aeruginosa*: dynamics, mechanisms, fitness trade-off and impact on *in vivo* efficacy. J Antimicrob Chemother 76:2546–2557. doi:10.1093/jac/dkab213. 34219168

[67] Masuda N, Sakagawa E, Ohya S, Gotoh N, Tsujimoto H, Nishino T. 2000. Substrate specificities of MexAB-OprM, MexCD-OprJ, and MexXY-oprM efflux pumps in *Pseudomonas aeruginosa*. Antimicrob Agents Chemother 44:3322–3327. doi:10.1128/AAC.44.12.3322-3327.2000. 11083635 PMC90200

[68] Klockgether J, Munder A, Neugebauer J, Davenport CF, Stanke F, Larbig KD, Heeb S, Schöck U, Pohl TM, Wiehlmann L, Tümmler B. 2010. Genome diversity of *Pseudomonas aeruginosa* PAO1 laboratory strains. J Bacteriol 192:1113–1121. doi:10.1128/JB.01515-09. 20023018 PMC2812968

[69] Chandler CE, Horspool AM, Hill PJ, Wozniak DJ, Schertzer JW, Rasko DA, Ernst RK. 2019. Genomic and phenotypic diversity among ten laboratory isolates of. J Bacteriol 201:e00595-18. doi:10.1128/JB.00595-18. 30530517 PMC6379574

[70] CLSI. Clinical and Laboratory Standards Institute. 2019. Performance standards for antimicrobial susceptibility testing; twenty-seventh informational supplement. CLSI document M100-S29. Clinical and Laboratory Standards Institute. Wayne, Pennsylvania.

[71] Nichols WW. 2017. Modeling the kinetics of the permeation of antibacterial agents into growing bacteria and its interplay with efflux. Antimicrob Agents Chemother 61:e02576-16. doi:10.1128/AAC.02576-16. 28717042 PMC5610481

