# Supplementary material for: Penicillin-Binding Protein 5/6 Acting as a Decoy Target in Pseudomonas aeruginosa Identified by Whole-Cell Receptor Binding and Quantitative Systems Pharmacology
Source: Antimicrob Agents Chemother. 2023 May 18;67(6):e01603-22. doi: 10.1128/aac.01603-22 (PMC10269149; doi:10.1128/aac.01603-22)
Supplement: Supplemental file 1 — Supplemental material. Download aac.01603-22-s0001.pdf, PDF file, 2.8 MB [file aac.01603-22-s0001.pdf]

Supplementary data for

Silvia López-Argüello et al.: Penicillin-Binding Protein 5/6 acting as a Decoy Target in *Pseudomonas aeruginosa* identified by Whole-Cell Receptor Binding and Quantitative Systems Pharmacology

METHODS

Principal component analyses (**PCA**) of the unbound fractions of each penicillin-binding protein (**PBP**) in intact and lysed bacteria were performed for all studied compounds using the XLSTAT software (version v2020.1). The unbound fractions at all time-points were analyzed on $\log_2$ scale. To empirically identify compounds with similar PBP occupancy patterns (i.e. similar sets of PBPs inactivated), compounds were compared based on their positions on the first two eigenvectors in intact and in lysed cells, respectively.

Additional Quantitative and Systems Pharmacology (QSP) modeling methods

Dependent variables and residual error model: We simultaneously fit the number of unbound PBP molecules per cell for each of the six different PBPs. An additive plus proportional residual error model was used with separate residual error parameters for each PBP. To account for a small degree of background noise in the SDS-PAGE gels, a noise parameter (e.g. Noise_{1a}) was incorporated for each PBP. This noise parameter defines the fraction of the Bocillin^{TM} FL band intensity which could not be inhibited (i.e. suppressed) even by a β-lactam with high affinity for the respective PBP at 2x the MIC. The noise parameters were constrained between 0 and 1, and described by a logistic transformation (i.e. a normal distribution on logistically transformed scale). All other parameters (i.e. the rate of net influx and PBP access, the acylation rate constants, and the initial conditions) were described by a log-normal distribution. The initial condition ($\mathbf{N_{PBP1a,0h}}$) for the number of unbound PBP1a molecules per cell was:

$$N_{PBP1a,0h} = 153 \cdot (1 - \text{Noise}_{1a}) \cdot \text{Fini}_{1a} \qquad (1)$$

A Noise_{1a} of 0.0 would indicate that the entire Bocillin^{TM} FL band intensity signal could be inhibited by a high affinity β-lactam at 2x MIC, whereas a Noise_{1a} value of 0.2 indicates that even a high affinity β-lactam would leave 20% of background signal. The same equation with separate parameters and initial numbers of PBPs was used for the other PBPs. The observed signal for PBP1a (Yobs_{PBP1a}) accounted for the background noise component via the second part of this output equation:

$$\text{Yobs}_{PBP1a} = N_{PBP1a} + 153 \cdot \text{Noise}_{1a} \cdot \text{Fini}_{1a} \qquad (2)$$

Initial conditions and output equations for the other PBPs used the same equations with separate parameters for the noise and initial condition, as well as the number of molecules for the respective PBP at 0 h. The complete population modeling estimation code is shown in **Figure S8**.

Dataset composition: Modeling all six dependent variables simultaneously required estimation of the rate of net influx and PBP access, as well as of the six acylation rate constants per drug, in addition to the six initial conditions and six noise parameters. To make this computationally more tractable, we created datasets with imipenem as 'backbone' drug that bound the largest set of PBPs and thus allowed sound estimation of the background noise parameters. To allow robust and efficient estimation, we elected to create datasets of four drugs each (i.e. imipenem plus three other drugs). Each of these datasets had 40 estimated population mean model parameters and twelve residual error parameters. Estimation of all 15 drugs simultaneously would have required the simultaneous estimation of over 100 model parameters (i.e. population means) which would have slowed down the

estimation substantially. Thus, performing a large number of iterations on datasets containing 4 drugs each, and using imipenem as backbone drug for each dataset, was more efficient.

Estimation. The QSP model parameters were estimated via population modelling in the S-ADAPT software (version 1.57, importance sampling algorithm, pmethod=4) (1-3). Competing models were evaluated based on the biological plausibility of the parameter estimates, the S-ADAPT objective function value (-1× log-likelihood), standard diagnostic plots, the coefficient of correlation and visual predictive checks (4-6).

Results

At the tested initial inoculum of approximately 7.6 log_{10} (CFU/mL), all β-lactams showed stasis (i.e. neither growth nor killing) during the first 60 min, except for imipenem which yielded approximately 1.5 log_{10} killing (**Fig. S1**). The β-lactamase inhibitors at 4 mg/L paralleled the growth control (results not shown). The SDS-PAGE gels for the PBP binding assay in intact (**Fig. S2**) and lysed cells (**Fig. S3**) showed overall more PBP inactivation in lysed compared to that in intact cells. Of note, imipenem and cefoxitin bound PBPs extensively both in intact and lysed cells.

The integrated band intensities for each PBP and drug were normalized against the PBP band intensity of the growth control at the respective time point. **Figure S4** shows the normalized band intensities for intact cells and **Figure S5** for lysed cells. A value of 0.0 would represent complete PBP inactivation, and a value of 1.0 no binding of the respective PBP. These data were normalized to 1.0 at 0 h for each PBP, and they represent the same raw data obtained from integrating the SDS-PAGE gels (**Fig. S2** and **S3**) that were also used for modeling (**Fig. 3** and **Fig. S7**). In the latter modeling plots, the data were expressed as number of unbound PBPs per cell instead of normalized to a baseline of 1.0 at 0 h.

We performed a principal component analysis (PCA) to group the compounds according to their PBP occupancy patterns in intact and lysed bacteria (**Fig. S6**). This empirical analysis seeks to identify compounds with similar PBP occupancy patterns, but does not provide mechanistic insights. This PCA used the extents of PBP inactivation as multiple input data, and then identified the axis associated with the largest variation (i.e. variance) in this multiple dimensional space. This axis becomes the first eigenvector. Then, the PCA identified an orthogonal axis that is associated with the second largest variance. This process is repeated using orthogonal axes for all eigenvectors. In this analysis, the first two eigenvectors represented the axes associated with the most extensive differences in PBP binding in our dataset (i.e. the largest variance). Accordingly, these two axes allow one to distinguish the overall PBP occupancy patterns between the 15 drugs. These two eigenvectors explained 65.9% of the total variance in intact cells and 77.1% in the isolated membrane assay. These two plots are mainly used to identify groups of compounds that provided similar PBP occupancy patterns within the lysed and within the intact cell dataset. However, differences in the position of various compounds (e.g. which quadrant) should not be compared between intact and lysed cells.

The complete estimation code of the QSP model with six dependent variables is shown in **Fig. S8**. In lysed cells, the outer membrane penetration step is lacking and a large number of drug molecules (10^7) is available to bind to the different PBPs. For intact cells, drugs have to first penetrate the outer membrane in order to compete for the binding of up to six different PBPs. This set of coupled differential equations implements the mass balance in the periplasmic space of *P. aeruginosa*.

Tables S1 shows the estimated second-order acylation rate constants for each of the PBPs and drugs. We performed sensitivity analyses for each dataset to select fixed values for the second-order acylation rate constants towards a high affinity target. For example, the second-order acylation rate constants of carbapenems for PBP2 were fixed to $500 \cdot 10^{-3}$ min^{-1}. Higher values were beneficial for some of the penicillins and cephalosporins (e.g. for PBP3), as shown in **Table S1**. With the first

observation being at 15 min, fixing the second-order acylation rate constants towards the highest affinity targets supported the estimation of the values of the rate constants to other PBPs.

Table S2 lists additional estimated population model parameters. The entire dataset of 15 drugs was split into five datasets with 4 drugs each. Cefoxitin was part of datasets 2 and 5, and the final estimates for cefoxitin arise from dataset 5, which contained three cephalosporins. The population means of the random multipliers of the initial condition (Fini) were close to 1.0, as expected. For datasets 1 and 2, the background noise for PBP3 was slightly higher, since the lysed cell assay data for the drugs in these datasets did not show complete PBP3 inactivation.

Table S3 compares the band intensities from SDS-PAGE gels between *Escherichia coli* (in-house data on file) and *P. aeruginosa* (present study). These results suggested that the assumption of a similar number of PBPs per cell between *P. aeruginosa* and *E. coli* is plausible. Even if this number of total expressed PBPs was slightly different in *P. aeruginosa*, this would not affect the relative comparison of drugs regarding their net penetration and access to PBPs in the present study.

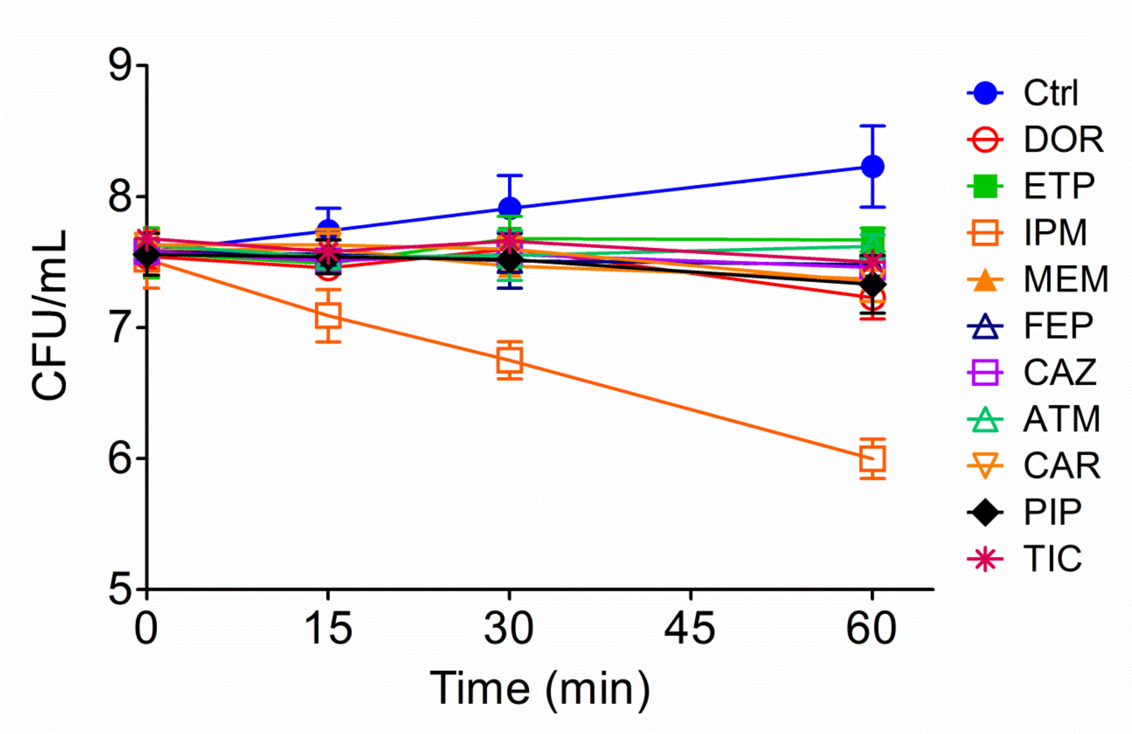

Figure S1. Static drug concentration time kill curves of *P. aeruginosa* PAO1 for ten β-lactams at 2x MIC. Drugs were doripenem (DOR; concentration studied: 2 mg/L), ertapenem (ETP; 8 mg/L), imipenem (IPM; 2 mg/L), meropenem (MEM; mg/L), cefepime (FEP; 2 mg/L), cefoxitin (FOX; 2,048 mg/L), ceftazidime (CAZ; 2 mg/L), aztreonam (ATM; 8 mg/L), carbenicillin (CAR; 96 mg/L), piperacillin (PIP; 8 mg/L) and ticarcillin (TIC; 48 mg/L). The average values from at least three experiments ± standard deviations are shown.

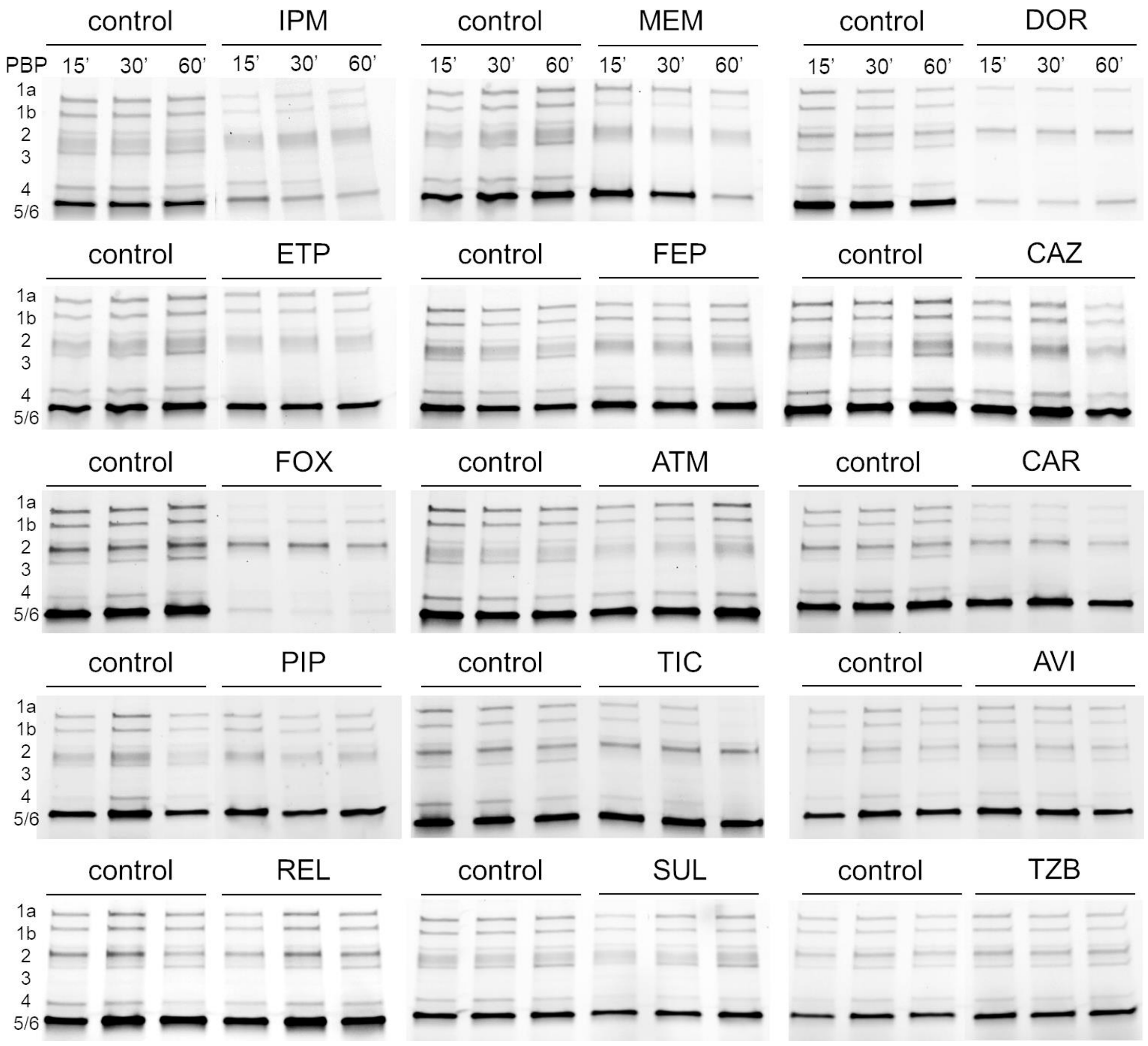

Figure S2. Gels for the whole cell PBP-binding assay for *P. aeruginosa* PAO1 over 60 min (data from one representative replicate shown). Within each replicate, serial samples were taken from the same culture at 15, 30 and 60 min. Drugs used were imipenem (IPM), meropenem (MEM), doripenem (DOR), ertapenem (ERT), cefepime (FEP), ceftazidime (CAZ), cefoxitin (FOX), aztreonam (ATM), carbenicillin (CAR), piperacillin (PIP), ticarcillin (TIC), avibactam (AVI), relebactam (REL), sulbactam (SUL) and tazobactam (TZB). The antibiotic-bound PBP-containing membrane preparations (protein concentration: 0.5 mg/mL) were collected by ultracentrifugation. Unbound PBPs were labelled with 25 µM Bocillin FL^{TM}. Antibiotic concentrations used were (2 × MIC): IPM = 2 mg/L; MEM = 1 mg/L; DOR = 2 mg/L; ETP = 8 mg/L; FEP = 2 mg/L; CAZ = 2 mg/L; FOX = 2,048 mg/L; ATM = 8 mg/L; CAR = 96 mg/L; PIP = 8 mg/L; TIC = 48 mg/L. The BLIs AVI, REL, SUL and TZB were studied at a fixed concentration of 4 mg/L. Labeled PBPs were separated through SDS-polyacrylamide gels and visualized using a FluorImager (excitation at 488 nm and emission at 530 nm).

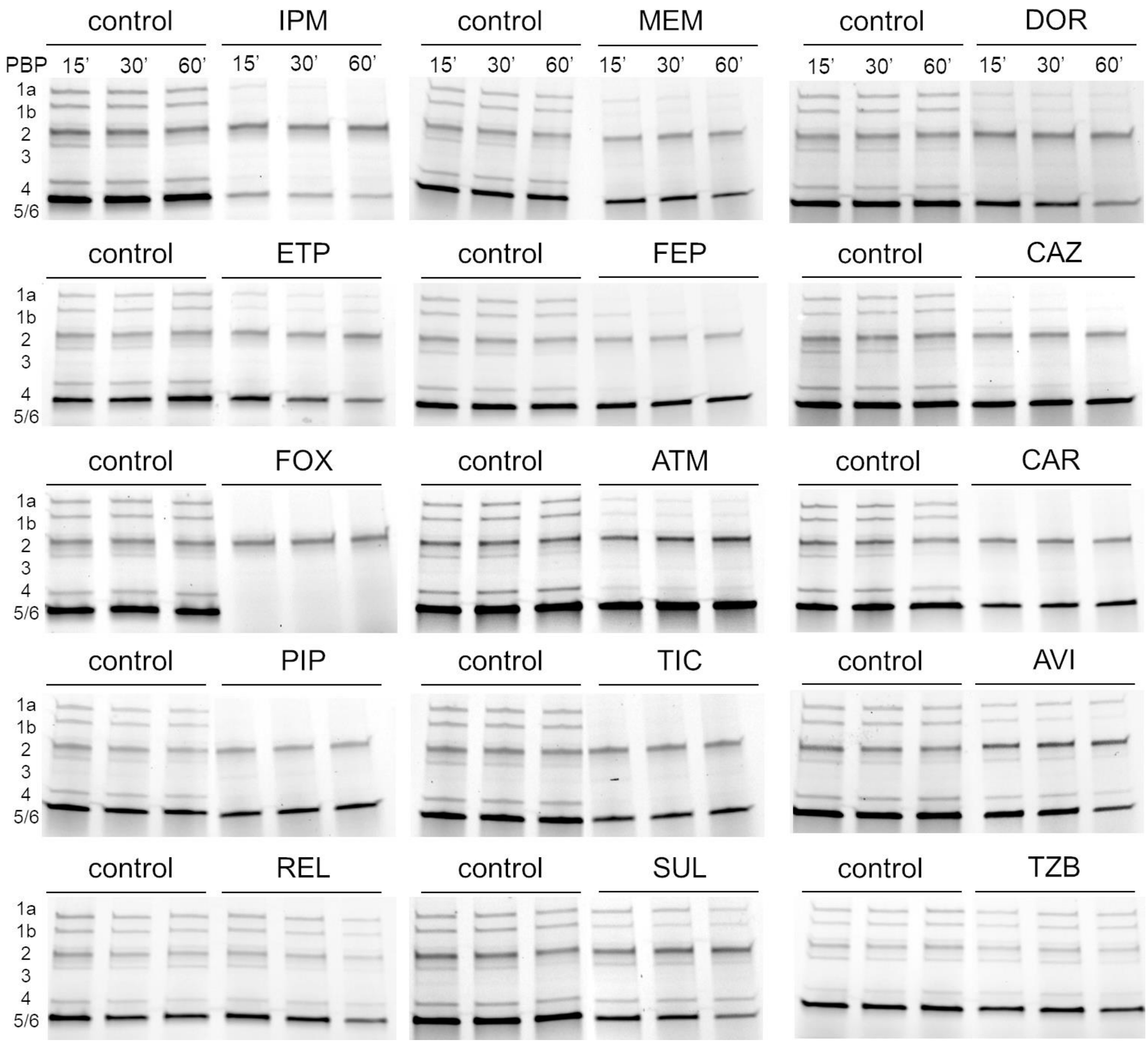

Figure S3. Gels for lysed cell (i.e., using isolated membranes) PBP-binding assay for *P. aeruginosa* PAO1 over 60 min (data from one representative replicate shown). Within each replicate, serial samples were taken from the same culture at 15, 30 and 60 min. The antibiotic-bound PBP-containing membrane preparations (protein concentration: 0.5 mg/mL) to label unbound PBPs with 25 μM Bocillin FL^{TM}. Drug concentrations and abbreviations are shown in **Figure S2**. Labeled PBPs were separated through SDS-polyacrylamide gels and visualized using a FluorImager (excitation at 488 nm and emission at 530 nm).

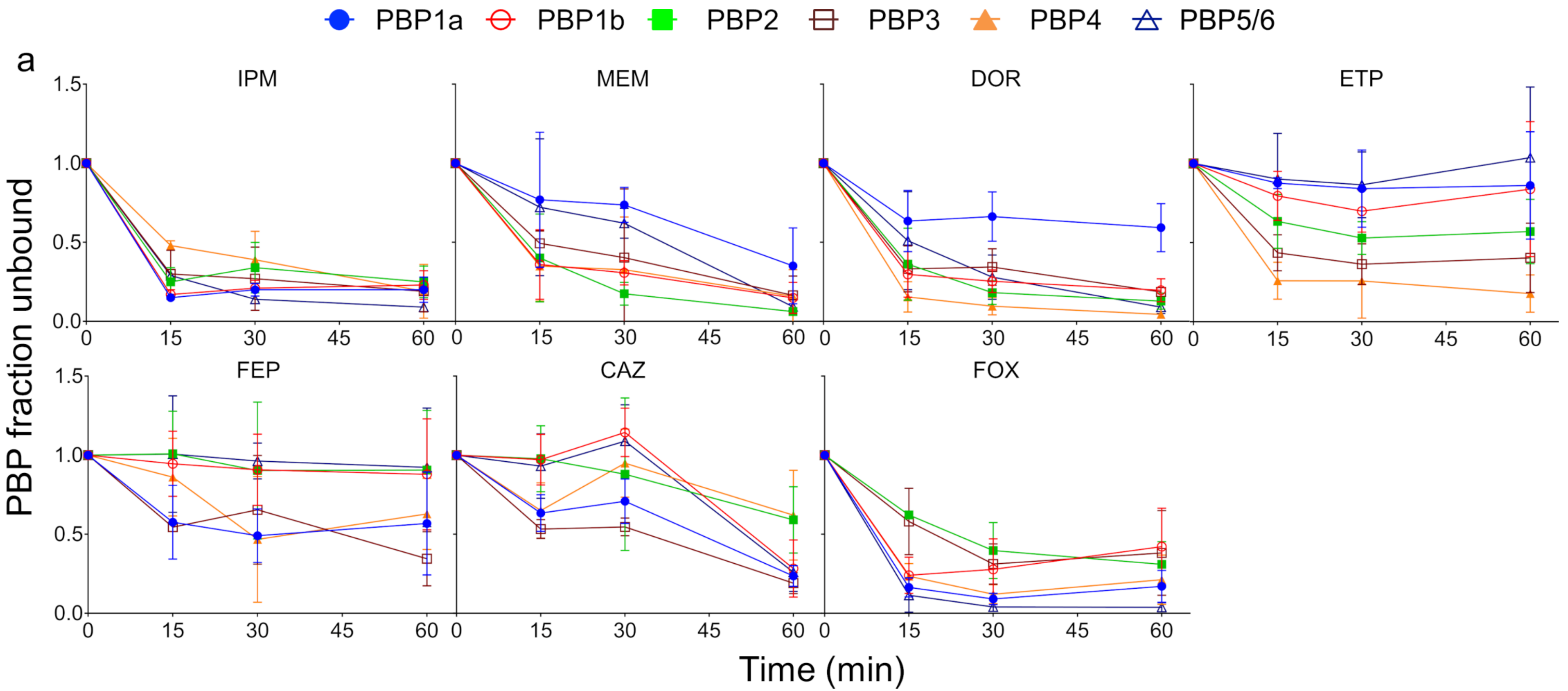

Figure S4. a Time-course of **whole cell** PBP-binding. *P. aeruginosa* PAO1 cultures were incubated for 15, 30 and 60 min in the presence of imipenem (IPM), meropenem (MEM), doripenem (DOR), ertapenem (ERT), cefepime (FEP), ceftazidime (CAZ), and cefoxitin (FOX). After isolating PBP-containing membranes, preparations were labeled with 25 μM Bocillin FL^{TM}. Antibiotic concentrations tested were 2x MIC: IPM = 2 mg/L; MEM = 1 mg/L; DOR = 2 mg/L; ETP = 8 mg/L; FEP = 2 mg/L; CAZ = 2 mg/L; FOX = 2,048 mg/L. The mean fraction of unbound PBPs from at least three biological replicates ± standard deviations are shown.

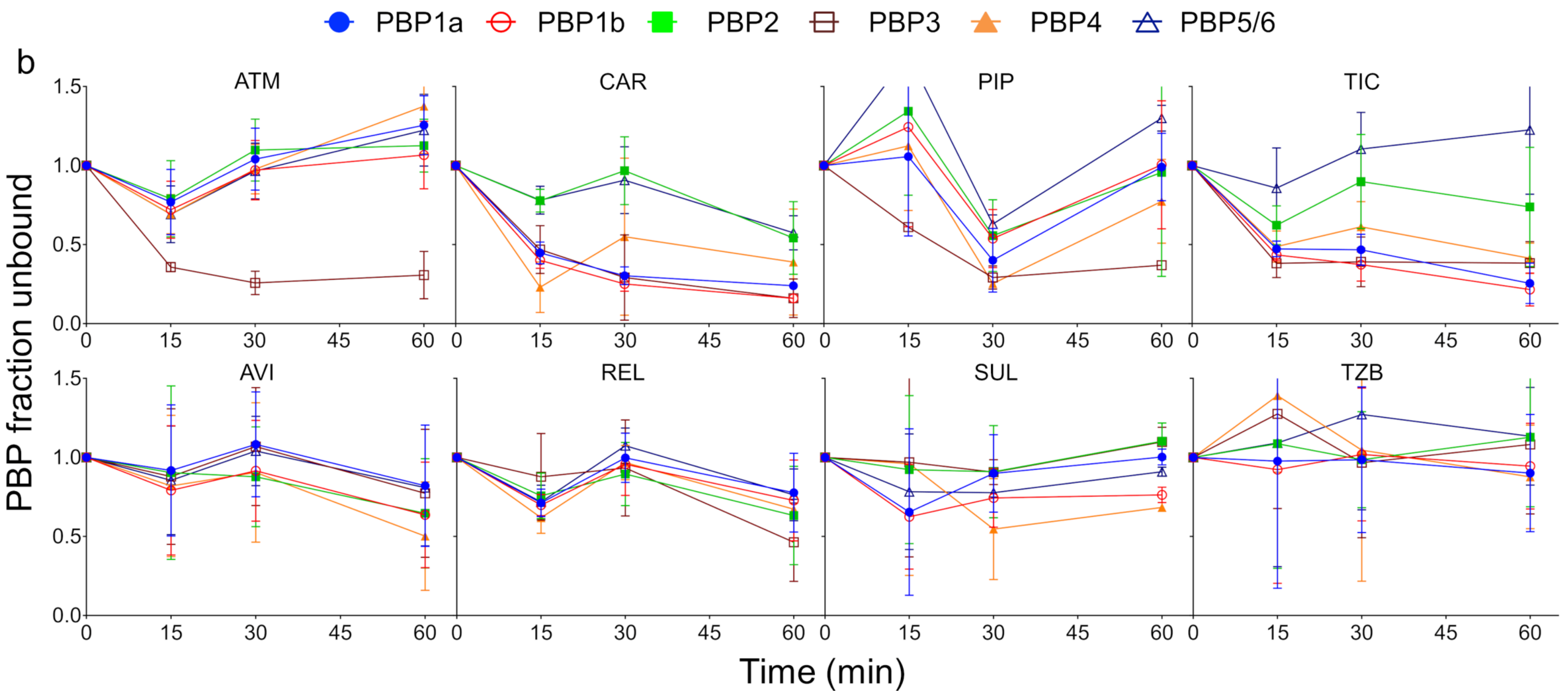

Figure S4. b Time-course of **whole cell** PBP-binding. *P. aeruginosa* PAO1 cultures were incubated for 15, 30 and 60 min in the presence of aztreonam (ATM), carbenicillin (CAR), piperacillin (PIP), ticarcillin (TIC), avibactam (AVI), relebactam (REL), sulbactam (SUL) and tazobactam (TZB). After isolating PBP-containing membranes, preparations were labeled with 25 μM Bocillin FL™. Antibiotic concentrations tested were 2x MIC: ATM = 8 mg/L; CAR = 96 mg/L; PIP = 8 mg/L; TIC = 48 mg/L. BLIs AVI, REL, SUL and TZB were used at a fixed concentration of 4 mg/L. The mean fraction of unbound PBPs from at least three biological replicates ± standard deviations are shown. Avibactam data were reproduced from our previous work Lopez-Argüello et al (7).

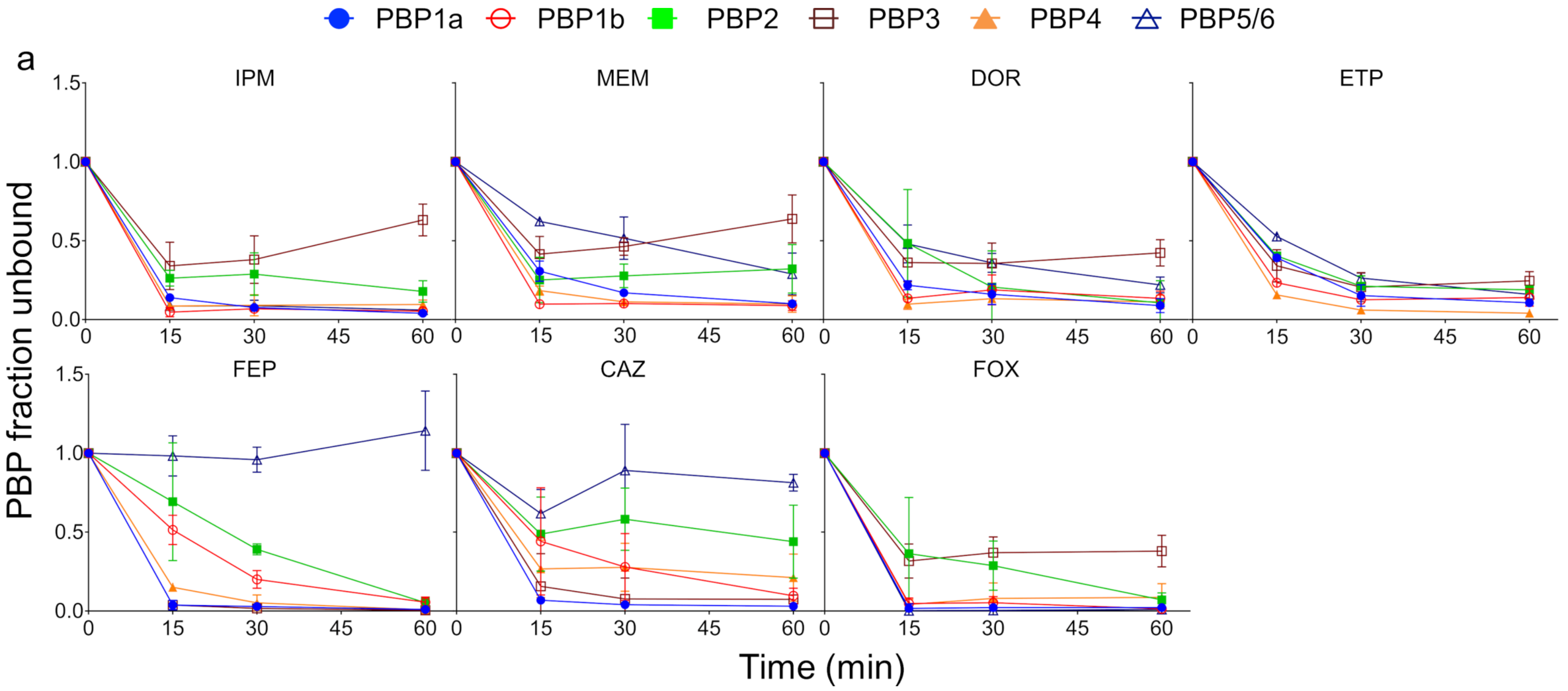

Figure S5. a Time-course of PBP-binding in **lysed cells** (i.e., using isolated membrane fractions). *P. aeruginosa* PAO1 PBP-containing membrane preparations were incubated for 15, 30 and 60 min in the presence of imipenem (IPM), meropenem (MEM), doripenem (DOR), ertapenem (ERT), cefepime (FEP), ceftazidime (CAZ), and cefoxitin (FOX). After isolating PBP-containing membranes, preparations incubated in the presence of the aforementioned drugs and labeled with 25 μM Bocillin FL^{TM}. Antibiotic concentrations tested were 2x MIC: IPM = 2 mg/L; MEM = 1 mg/L; DOR = 2 mg/L; ETP = 8 mg/L; FEP = 2 mg/L; CAZ = 2 mg/L; FOX = 2,048 mg/L. The average values from at least biological replicates ± standard deviations are shown.

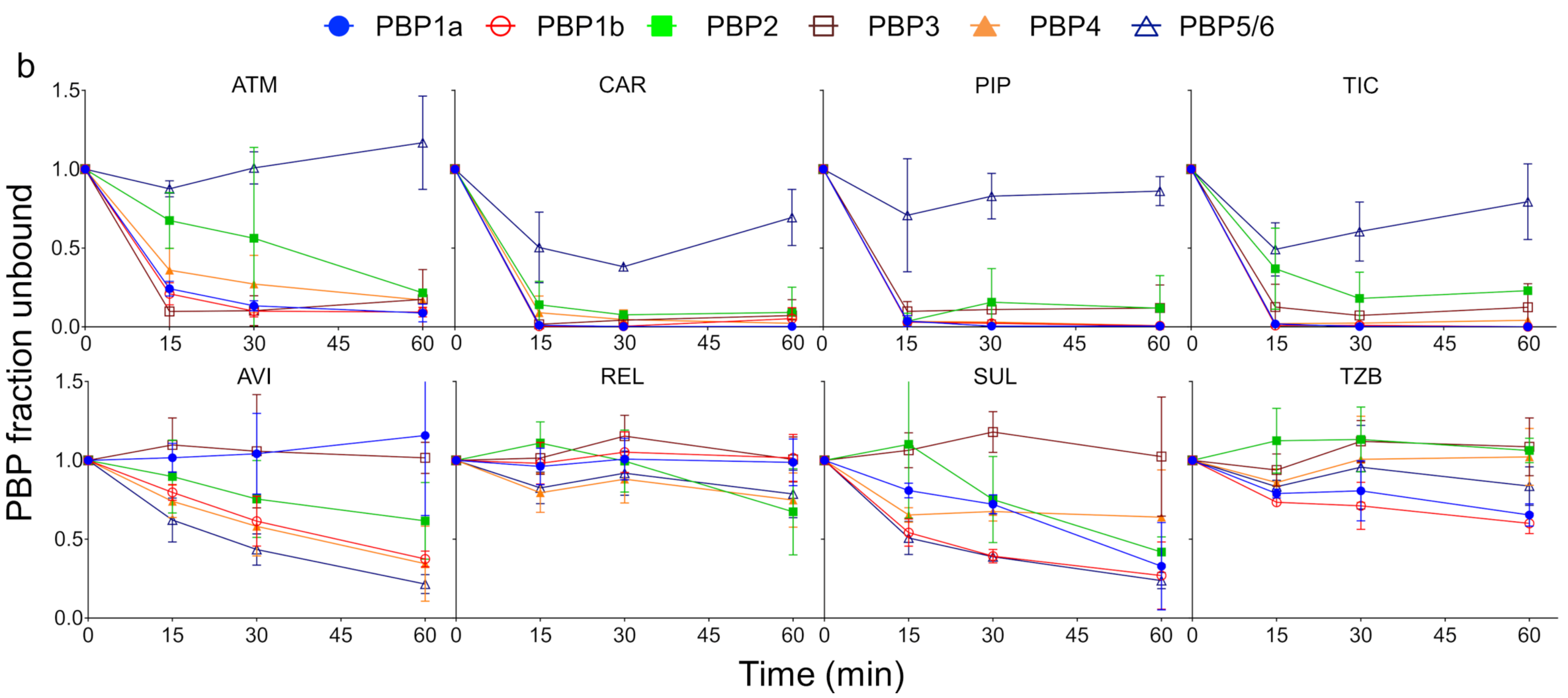

Figure S5. b Time-course of PBP-binding in lysed cells (i.e., using isolated membrane fractions). *P. aeruginosa* PAO1 PBP-containing membrane preparations were incubated for 15, 30 and 60 min in the presence of aztreonam (ATM), carbenicillin (CAR), piperacillin (PIP), ticarcillin (TIC), avibactam (AVI), relebactam (REL), sulbactam (SUL) and tazobactam (TZB). After isolating PBP-containing membranes, preparations were labeled with 25 μM Bocillin FL^{TM}. Antibiotic concentrations tested were 2x MIC: ATM = 8 mg/L; CAR = 96 mg/L; PIP = 8 mg/L; TIC = 48 mg/L. BLIs AVI, REL, SUL and TZB were used at a fixed concentration of 4 mg/L. The average values from at least biological replicates ± standard deviations are shown. Avibactam data reproduced from our previous work Lopez-Argüello et al (7).

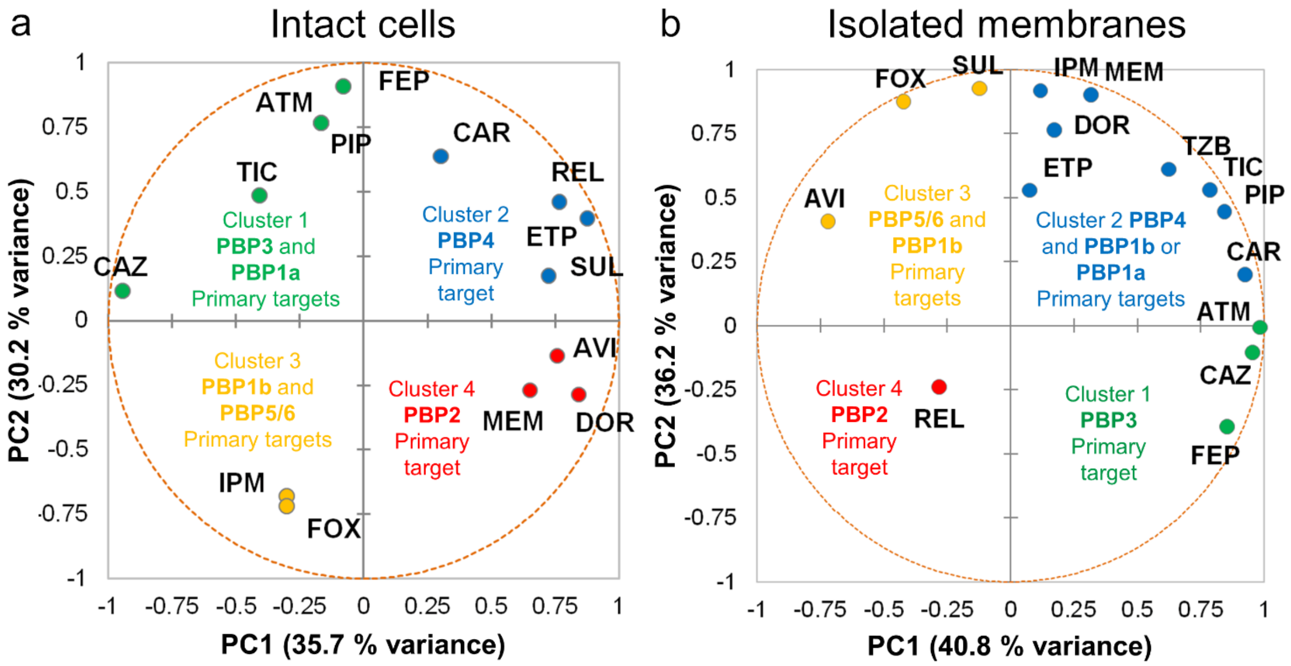

Figure S6. Principal component analysis (**PCA**) of the log-transformed unbound fractions of PBPs for 15 drugs (a) in intact cells (over all time-points) and (b) isolated membranes of *P. aeruginosa* PAO1. Compounds were empirically grouped into four general clusters based on their positions on the first and second eigenvector. Some drugs were grouped into different clusters between the lysed and intact cell datasets, due to their poor target site penetration. DOR, doripenem; ETP, ertapenem; IPM, imipenem; MEM, meropenem; FEP, cefepime; FOX, cefoxitin; CAZ, ceftazidime; ATM, aztreonam; CAR, carbenicillin; PIP, piperacillin; TIC, ticarcillin; AVI, avibactam; REL, relebactam; SUL, sulbactam; TZB, tazobactam. To strengthen the robustness of our analysis, previously obtained data on avibactam were included as additional biological replicates.

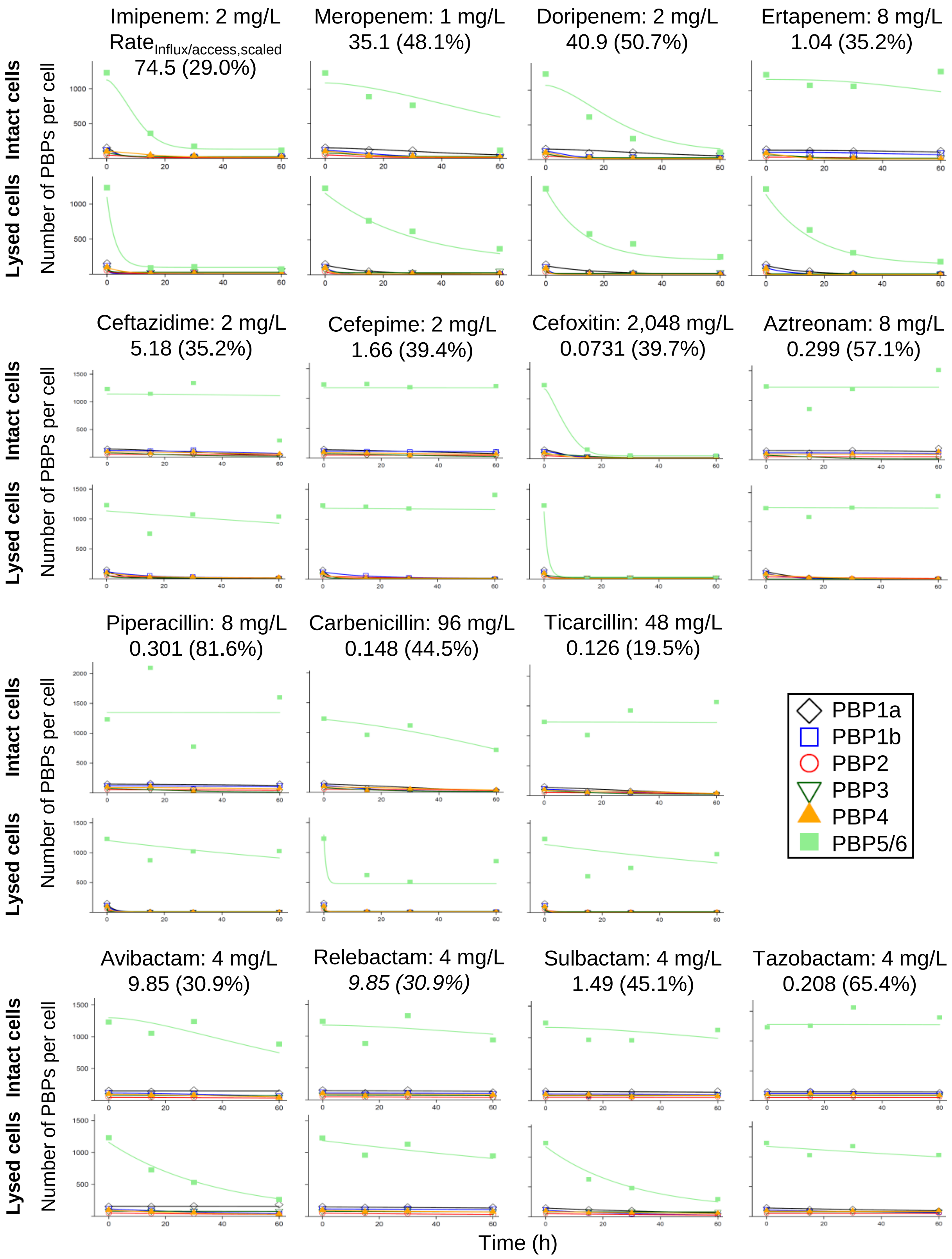

Figure S7. Time-course for the binding of six PBPs in intact (upper panels) and lysed cells (lower panels) of *P. aeruginosa* PAO1. Data are averages of two or three biological replicates. The studied drug concentrations are indicated for each compound, along with the estimate for the scaled rate of net influx and PBP access ($Rate_{Influx/access,scaled}$) from population modeling (along with its relative standard error; SE%). The $Rate_{Influx/access,scaled}$ has a unit of drug molecules per min per mg/L of extracellular drug concentration. The PBP5/6 had the highest expression (i.e. number of PBP molecules per bacterial cell).

Figure S8. Estimation code for the final model (using the dataset with imipenem, aztreonam, piperacillin and carbenicillin as an example) in SADAPT-TRAN format

```
$PROJECT THIS WILL BE A TERRIFIC PROJECT

$DIFFEQ_DIF

PBP1a  = X(1) ; number of PBP1a  per cell
PBP1b  = X(2) ; number of PBP1b  per cell
PBP2   = X(3) ; number of PBP2   per cell
PBP3   = X(4) ; number of PBP3   per cell
PBP4   = X(5) ; number of PBP4   per cell
PBP5   = X(6) ; number of PBP5/6 per cell

Nperi  = X(7) ; number of drug molecules in periplasm per cell

MM1a = K1a*Nperi/(KM_1a+Nperi)
MM1b = K1b*Nperi/(KM_1b+Nperi)
MM2  = K2 *Nperi/(KM_2 +Nperi)
MM3  = K3 *Nperi/(KM_3 +Nperi)
MM4  = K4 *Nperi/(KM_4 +Nperi)
MM5  = K5 *Nperi/(KM_5 +Nperi)

XP(1)= - MM1a*PBP1a
XP(2)= - MM1b*PBP1b
XP(3)= - MM2 *PBP2
XP(4)= - MM3 *PBP3
XP(5)= - MM4 *PBP4
XP(6)= - MM5 *PBP5

XP(7)= NET_INFLUX_PBP_ACCESS_RATE - MM1a*PBP1a - MM1b*PBP1b - MM2*PBP2 - MM3*PBP3 - MM4*PBP4 - MM5*PBP5

$OUTPUT_GLB

IF (DRUGNO.EQ.1) THEN
   Rinflux = Rinf_IPM
   K2_1A   = K2_1A_IPM
   K2_1B   = K2_1B_IPM
   K2_2    = K2_2_IPM
   K2_3    = K2_3_IPM
   K2_4    = K2_4_IPM
   K2_5    = K2_5_IPM
ELSEIF (DRUGNO.EQ.8) THEN
   Rinflux = Rinf_ATM
   K2_1A   = K2_1A_ATM
   K2_1B   = K2_1B_ATM
   K2_2    = K2_2_ATM
   K2_3    = K2_3_ATM
   K2_4    = K2_4_ATM
   K2_5    = K2_5_ATM
ELSEIF (DRUGNO.EQ.9) THEN
   Rinflux = Rinf_PIP
   K2_1A   = K2_1A_PIP
   K2_1B   = K2_1B_PIP
   K2_2    = K2_2_PIP
   K2_3    = K2_3_PIP
   K2_4    = K2_4_PIP
   K2_5    = K2_5_PIP
ELSE
   Rinflux = Rinf_CAR
   K2_1A   = K2_1A_CAR
   K2_1B   = K2_1B_CAR
   K2_2    = K2_2_CAR
   K2_3    = K2_3_CAR
   K2_4    = K2_4_CAR
   K2_5    = K2_5_CAR
ENDIF
```

Figure S8. Estimation code for the final model (using the dataset with imipenem, aztreonam, piperacillin and carbenicillin as an example) in SADAPT-TRAN format – **continued**

```
IF (INTACT.EQ.1) THEN
   NET_INFLUX_PBP_ACCESS_RATE = Rinflux*CDRUG  ; number of drug molecules per minute - normalized by extracellular drug conc.
   N_IN_IC                    = 0              ; number of drug molecules in periplasm - zero for intact cell experiments
ELSE
   NET_INFLUX_PBP_ACCESS_RATE = 0        ; number of drug molecules per minute
   N_IN_IC                    = 10000000 ; number of drug molecules at the lysed cell target site
                                         ; 10 million molecules just assumed as a large excess
ENDIF

K1a = K2_1a/1000
K1b = K2_1b/1000
K2  = K2_2 /1000
K3  = K2_3 /1000
K4  = K2_4 /1000
K5  = K2_5 /1000

$OUTPUT_ICS

X(1) = X(1) +  153*(1-Noise_1a)*Fini_1A
X(2) = X(2) +  118*(1-Noise_1b)*Fini_1B
X(3) = X(3) +   50*(1-Noise_2) *Fini_2
X(4) = X(4) +   79*(1-Noise_3) *Fini_3
X(5) = X(5) +   99*(1-Noise_4) *Fini_4
X(6) = X(6) + 1232*(1-Noise_5) *Fini_5
X(7) = X(7) + N_IN_IC

$OUTPUT_EQN

IF (X(1).LT.0) X(1)= 0
IF (X(2).LT.0) X(2)= 0
IF (X(3).LT.0) X(3)= 0
IF (X(4).LT.0) X(4)= 0
IF (X(5).LT.0) X(5)= 0
IF (X(6).LT.0) X(6)= 0
IF (X(7).LT.0) X(7)= 0

Y(1)  = X(1) +  153*Noise_1a*Fini_1A
Y(2)  = X(2) +  118*Noise_1b*Fini_1B
Y(3)  = X(3) +   50*Noise_2 *Fini_2
Y(4)  = X(4) +   79*Noise_3 *Fini_3
Y(5)  = X(5) +   99*Noise_4 *Fini_4
Y(6)  = X(6) + 1232*Noise_5 *Fini_5

$VARMOD_EQN

V(1) = ( SDIN1a + SDSL1a * Y(1) ) * ( SDIN1a + SDSL1a * Y(1) )
V(2) = ( SDIN1b + SDSL1b * Y(2) ) * ( SDIN1b + SDSL1b * Y(2) )
V(3) = ( SDIN2  + SDSL2  * Y(3) ) * ( SDIN2  + SDSL2  * Y(3) )
V(4) = ( SDIN3  + SDSL3  * Y(4) ) * ( SDIN3  + SDSL3  * Y(4) )
V(5) = ( SDIN4  + SDSL4  * Y(5) ) * ( SDIN4  + SDSL4  * Y(5) )
V(6) = ( SDIN5  + SDSL5  * Y(6) ) * ( SDIN5  + SDSL5  * Y(6) )

$POPMOD_EQN
```

Table S1. Second-order acylation rate constants (unit: 10^{-3} min^{-1}) for each PBP and 15 drugs.

**Drug**	Imipenem	Doripenem	Meropenem	Ertapenem	Ceftazidime	Cefepime	Cefoxitin
PBP1a	2,210	49.3	74.5	82.4	218	396	864
PBP1b	1,710	677	307	133	80.6	68.7	795
PBP2	500 [a]	500 [a]	500 [a]	500 [a]	67.6	51.7	495
PBP3	990	1350	449	8,400	563	1,000 [a]	240
PBP4	189	878	529	18,700	69.3	252	1,000 [a]
PBP5/6	335	85.7	34.0	61.8	3.51	0.291	767

**Drug**	Aztreonam	Piperacillin	Carbenicillin	Ticarcillin	Avibactam	Relebactam	Sulbactam	Tazobactam
PBP1a	100	456	3,030	1,840	0.163	2.41	14.9	7.02
PBP1b	128	489	9,520	2,200	23.0	1.69	27.4	9.17
PBP2	16.7	628	984	462	15.1 [b]	15.1 [b]	16.5	0.263
PBP3	10,000 [a]	10,000 [a]	10,000 [a]	5,000 [a]	0.923	3.2	0.317	0.552
PBP4	46.2	642	1580	839	26.3	5.93	8.38	0.694
PBP5/6	0.144	6.82	1270	5.90	24.5	4.72	33.5	3.00

[a]: These second-order acylation rate constants for high affinity targets were eventually fixed after thorough sensitivity analyses of multiple values for each dataset.

[b]: The apparent acylation rate constant for PBP2 by relebactam was shared with the estimate of avibactam, since relebactam displayed limited binding.

Table S2. Additional parameter estimates of the population model in the four datasets (each dataset contained IPM as a backbone)

Parameter	Dataset 1<br>IPM, DOR, MEM, ETP	Dataset 2<br>IPM, AVI, REL, (FOX)	Dataset 3<br>IPM, ATM, PIP, CAR	Dataset 4<br>IPM, TZB, SUL, TIC	Dataset 5<br>IPM, CAZ, FEP, FOX
Fraction of background noise intensity for each PBP band in gels					
NOISE_1a	0.112	0.0905	0.0659	0.08	0.0687
NOISE_1b	0.143	0.152	0.0775	0.0769	0.129
NOISE_2	0.236	0.287	0.157	0.242	0.287
NOISE_3	0.364	0.359	0.168	0.235	0.231
NOISE_4	0.139	0.125	0.0763	0.0346	0.122
NOISE_5	0.126	0.042	0.274	0.0878	0.0422
Multipliers on initial condition for the respective PBP					
Fini,1a	0.944	0.977	0.943	0.934	0.940
Fini,1b	0.949	0.966	0.972	0.919	1.01
Fini,2	0.942	0.983	0.981	1.00	0.990
Fini,3	0.945	0.973	0.950	1.02	0.949
Fini,4	0.982	0.951	0.898	0.888	0.929
Fini,5/6	0.923	0.979	1.00	0.973	0.943
Standard deviation of additive residual error					
SDin1a	4.60	4.91	4.73	5.21	3.40
SDin1b	2.13	9.89	3.05	2.40	10.3
SDin2	7.43	8.52	5.58	3.66	7.85
SDin3	13.1	6.87	9.68	8.92	13.1
SDin4	9.80	9.54	1.59	0.755	5.53
SDin5/6	3.30	22.7	156	6.23	17.9
Proportional residual error (i.e. coefficient of variation)					
SDsl1a	0.203	0.16	0.233	0.205	0.206
SDsl1b	0.194	0.0697	0.187	0.239	0.103
SDsl2	0.0251	0.0073	0.131	0.166	0.0428
SDsl3	0.103	0.135	0.0436	0.141	0.0838
SDsl4	0.0214	0.0881	0.380	0.371	0.189
SDsl5/6	0.295	0.162	0.106	0.266	0.239

All Noise and Fini parameters carry no unit (they are fractions). The standard deviations of the additive residual errors (SDin) carry units of number of PBP molecules per cell. The coefficient of variation of the proportional residual error (SDsl) carries no unit.

Table S3. Comparison of fluorescent band intensities from SDS-PAGE gels between *Escherichia coli* (in-house data on file) and *P. aeruginosa* (present study). Data are averages of n=6 replicates per pathogen.

	**Fluorescent band intensity** from SDS-PAGE gels		
**PBP**	***P. aeruginosa***	***E. coli***	**Ratio**
**1a**	951	885	1.07
**1b**	880	722	0.82
**2**	294	559	1.90
**3**	554	1,019	1.84
**4**	1,008	1,446	1.43
**5/6**	12,325	12,073	0.98
**Sum**	**16,012**	**16,704**	**0.96**

References

1. Bulitta JB, Bingolbali A, Shin BS, Landersdorfer CB. Development of a new pre- and post-processing tool (SADAPT-TRAN) for nonlinear mixed-effects modeling in S-ADAPT. The AAPS journal. 2011;13(2):201-11. Epub 2011/03/04. doi: 10.1208/s12248-011-9257-x. PubMed PMID: 21369876.
2. Bauer RJ, Guzy S, Ng C. A survey of population analysis methods and software for complex pharmacokinetic and pharmacodynamic models with examples. The AAPS journal. 2007;9(1):E60-83. PubMed PMID: 17408237.
3. Bulitta JB, Landersdorfer CB. Performance and robustness of the Monte Carlo importance sampling algorithm using parallelized S-ADAPT for basic and complex mechanistic models. The AAPS journal. 2011;13(2):212-26. Epub 2011/03/05. doi: 10.1208/s12248-011-9258-9. PubMed PMID: 21374103.
4. Bulitta JB, Duffull SB, Kinzig-Schippers M, Holzgrabe U, Stephan U, Drusano GL, et al. Systematic comparison of the population pharmacokinetics and pharmacodynamics of piperacillin in cystic fibrosis patients and healthy volunteers. Antimicrob Agents Chemother. 2007;51(7):2497-507. doi: 10.1128/AAC.01477-06. PubMed PMID: 17485505; PubMed Central PMCID: PMC1913222.
5. Tsuji BT, Okusanya OO, Bulitta JB, Forrest A, Bhavnani SM, Fernandez PB, et al. Application of pharmacokinetic-pharmacodynamic modeling and the justification of a novel fusidic acid dosing regimen: raising Lazarus from the dead. Clin Infect Dis. 2011;52 Suppl 7:S513-9. Epub 2011/05/13. doi: 10.1093/cid/cir166. PubMed PMID: 21546628.
6. Bulitta JB. Informing and Validating Translational Mechanism-Based Models for Antibiotics by Experimental and Computational Approaches. Clin Pharmacol Ther. 2021;110(6):1426-8. doi: 10.1002/cpt.2367. PubMed PMID: 34555178.
7. López-Argüello S, Montaner M, Oliver A, Moya B. Molecular Basis of AmpC β-Lactamase Induction by Avibactam in *Pseudomonas aeruginosa*: PBP Occupancy, Live Cell Binding Dynamics and Impact on Resistant Clinical Isolates Harboring PDC-X Variants. Int J Mol Sci. 2021;22(4):3051. doi: 10.3390/ijms22063051.
